# Transcriptomic Changes in Coral Holobionts Provide Insights into Physiological Challenges of Future Climate and Ocean Change

**DOI:** 10.1371/journal.pone.0139223

**Published:** 2015-10-28

**Authors:** Paulina Kaniewska, Chon-Kit Kenneth Chan, David Kline, Edmund Yew Siang Ling, Nedeljka Rosic, David Edwards, Ove Hoegh-Guldberg, Sophie Dove

**Affiliations:** 1 Australian Institute of Marine Science, PMB 3, Townsville MC, Queensland, Australia; 2 School of Biological Sciences, The University of Queensland, St Lucia, Queensland, Australia; 3 School of Agriculture and Food Sciences, The University of Queensland, St Lucia, Queensland, Australia; 4 Scripps Institution of Oceanography, University of California San Diego, La Jolla, California, United States of America; 5 University of Queensland Centre for Clinical Research, The University of Queensland, Herston, Queensland, Australia; 6 Global Change Institute and ARC Centre of Excellence for Coral Reef Studies, The University of Queensland, St Lucia, Queensland, Australia; Pennsylvania State University, UNITED STATES

## Abstract

Tropical reef-building coral stress levels will intensify with the predicted rising atmospheric CO_2_ resulting in ocean temperature and acidification increase. Most studies to date have focused on the destabilization of coral-dinoflagellate symbioses due to warming oceans, or declining calcification due to ocean acidification. In our study, pH and temperature conditions consistent with the end-of-century scenarios of the Intergovernmental Panel on Climate Change (IPCC) caused major changes in photosynthesis and respiration, in addition to decreased calcification rates in the coral *Acropora millepora*. Population density of symbiotic dinoflagellates (*Symbiodinium*) under high levels of ocean acidification and temperature (Representative Concentration Pathway, RCP8.5) decreased to half of that found under present day conditions, with photosynthetic and respiratory rates also being reduced by 40%. These physiological changes were accompanied by evidence for gene regulation of calcium and bicarbonate transporters along with components of the organic matrix. Metatranscriptomic RNA-Seq data analyses showed an overall down regulation of metabolic transcripts, and an increased abundance of transcripts involved in circadian clock control, controlling the damage of oxidative stress, calcium signaling/homeostasis, cytoskeletal interactions, transcription regulation, DNA repair, Wnt signaling and apoptosis/immunity/ toxins. We suggest that increased maintenance costs under ocean acidification and warming, and diversion of cellular ATP to pH homeostasis, oxidative stress response, UPR and DNA repair, along with metabolic suppression, may underpin why Acroporid species tend not to thrive under future environmental stress. Our study highlights the potential increased energy demand when the coral holobiont is exposed to high levels of ocean warming and acidification.

## Introduction

Changes in atmospheric CO_2_ are likely to fundamentally alter ocean ecosystems through their influence on sea temperature and carbonate ion chemistry [1,2]. Coral reef ecosystems are among the major oceanic systems that are likely to be detrimentally affected by global warming and ocean acidification (OA) [3,4]. These highly productive and biologically diverse ecosystems provide important goods and services to more than 450 million people in coastal communities around the world [5].

Local and global stressors are affecting corals and reef communities, and threaten their long-term survival [6]. Rapidly warming oceans threaten reef-building corals as anomalously high temperatures lead to the breakdown of symbiosis between the coral host and its symbiotic dinoflagellates, a phenomenon called coral “bleaching”. Over the past few decades, mass coral bleaching events have increased both in frequency and intensity [7–9]. The result of these events is usually high coral mortality and for colonies that survive, decreased colony growth and depressed reproductive output is common [10,11]. There are differences in bleaching susceptibility and severity among coral species and it has been shown that fast-growing branching genera such as *Acropora* are more susceptible to severe bleaching [8,12].

High oceanic uptake of increased atmospheric CO_2_ resulting in OA also poses threats to a range of marine organisms, as it can potentially affect rates of calcium carbonate deposition due to the reduction in the saturation state of carbonate forms such as aragonite, calcite and magnesium calcite [13]. Most of the experimental studies on the effects of OA on marine calcifiers have been conducted in tank or mesocosm experiments [4], with a few studies conducted in the field showing changes in coral species composition or shifts from hard to soft coral dominance [14,15]. Studies have shown that reef calcifiers, such as corals and calcifying algae, will have decreased rates of calcification under future OA conditions [16,17]. To date, studies on the effect of OA on the rate of calcification of marine calcifiers have tended to dominate the literature [16], with a strong negative correlation between calcification rates and OA [4,16,18,19]. Given the importance of variables such as pH and the carbonate ion concentration, it is perhaps not surprising that a large range of physiological processes appear to be influenced by OA in marine organisms [20–22] and there is evidence that OA can affect symbiont population density and depress metabolism, processes that lead to the biological deposition of calcium carbonate in reef-building corals, before effects on calcification rates are apparent [23].

To a large extent, most previous studies have examined ocean warming and ocean acidification in isolation of each other with notable exceptions [24]. Considering that future changes in atmospheric CO_2_ concentrations will affect both ocean temperature and chemistry at the same time, it is important to study the combined effects of temperature change and OA. Recently there has been an increase in reports on the combined effect of increased temperature and pCO_2_ on marine calcifiers [24–27]. Overall it is clear that responses are variable, can be nonlinear and there are intra- and inter-specific variances for reef-building corals, where the combined effect of high temperatures and high pCO_2_ levels mostly leads to decreases in calcification rates [24–27].

Previous studies have mainly focused on the effect of future changes in ocean temperature and chemistry on calcification, while many other physiological processes, such as photosynthesis, energy metabolism, changes to cell membrane physiology, reproduction, overall fitness and energy costs associated with acclimation to environmental conditions have received less attention [23]. In addition, there is a lack of knowledge about the transcriptional regulation of specific molecular pathways involved in changes in calcification, bleaching and stress response observed at the phenotype level, when exposed to changes in ocean temperature and chemistry. A few studies have investigated changes in global gene expression in response to OA in juvenile [28] and adult corals [23,29], and there is knowledge on the effect of increases in temperatures on both larval and adult coral transcriptomes [30–34]. To date, however, there is no information on the effect of different future scenarios of ocean warming and chemistry on the physiology of reef-building corals, specifically in terms of photosynthesis and respiration rates, *Symbiodinium* density and pigment concentrations, host protein and lipid levels, and calcification rates, coupled with the underlying molecular mechanisms for changes observed at the phenotype level.

The aim of our study was to contribute to this knowledge gap and investigate how changes in both temperature and pCO_2_ affect the physiology of the coral holobiont, and how the changes are reflected in the metatranscriptome. Branching Acroporid corals are important reef builders that create most of the habitat complexity in the Indo-Pacific, and have been found to be highly sensitive to both thermal and OA stress [8,12,18]. Based on these biological features we chose *Acropora millepora* as the model species for this study. In this study we aimed to measure changes in selected physiological processes in the coral holobiont, which highlight important biological functions such as photosynthesis (oxygen evolution rates, *Symbiodinium* pigment concentrations), respiration, coral-algal symbiosis (*Symbiodinium* population densities), energy storage potential (lipid and protein) and calcification. These selected physiological processes can provide an indication of overall coral holobiont health status and calcification potential of the organism when exposed to environmental stress.

## Results

### Selected physiological processes of the coral holobiont

**Fig 1:**
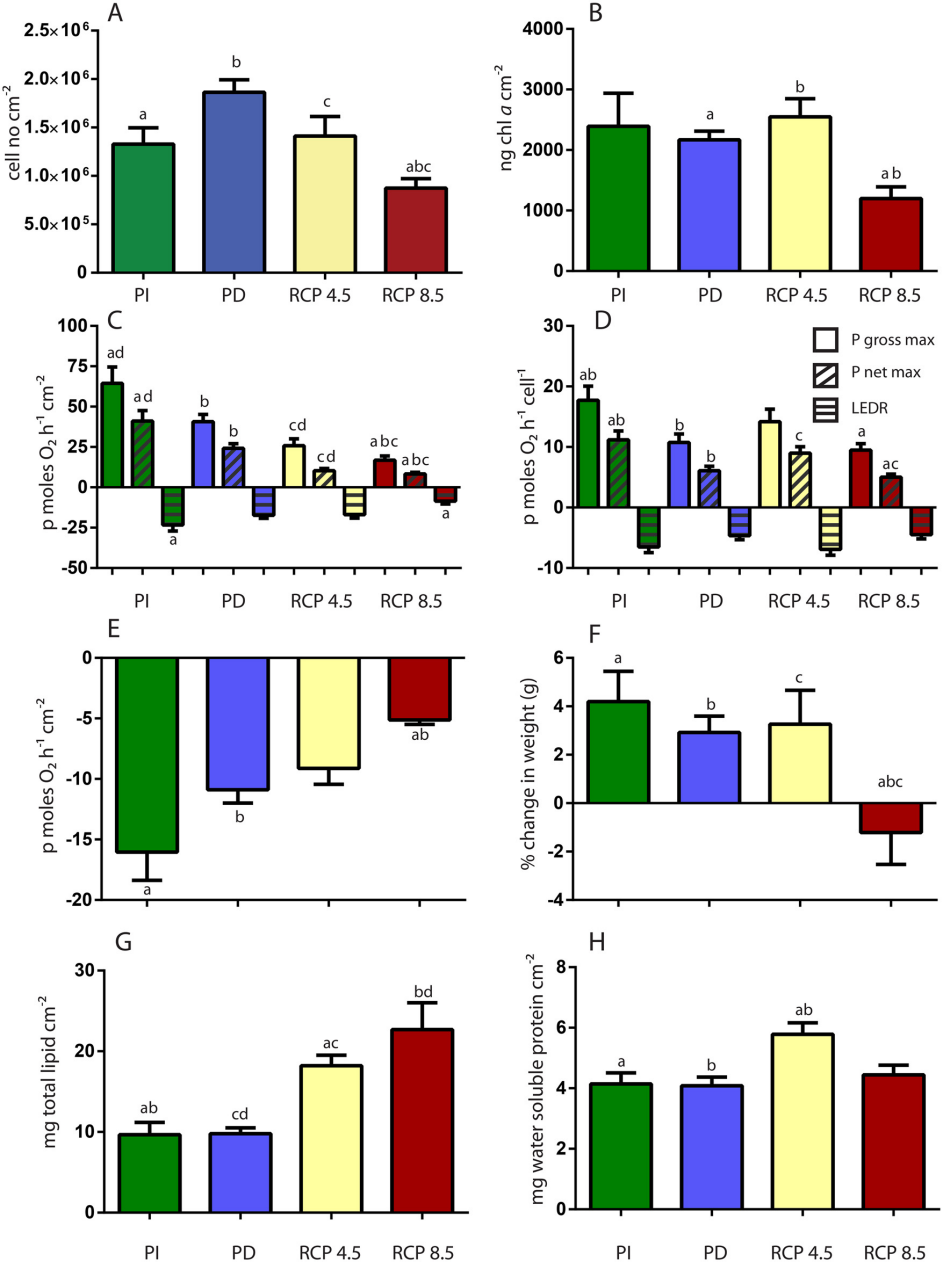
The effect of past and future changes in seawater temperature and pCO_2_ (Pre Industrial, PI, Present Day, PD, Representative Concentration Pathway RCP4.5 and RCP8.5) conditions on coral-algal physiology. (A) *Symbiodinium* cell number (B) chlorophyll *a* levels per surface area (C) photosynthetic capacity per surface area measured as P_net_ max, light enhanced dark respiration(LEDR) and P_gross_ max (P_net_ max—LEDR) (D) photosynthetic capacity per symbiont cell measured as P_net_ max, light enhanced dark respiration(LEDR) and P_gross_ max (P_net_ max—LEDR) (E) dark respiration (R_dark_) (F) relative calcification/growth as % change in weight (G) total lipid content and (H) total water soluble protein content. Error bars represent the standard error of the mean (n = 16). *Post hoc* differences are provided above the relevant data in letter symbols.

The physiology in terms of selected measured processes of the reef building coral, *A*. *millepora*, varied according to whether the coral was exposed to past, present-day or future scenarios of ocean temperatures and pCO_2_ (Table 1). Our results show that the *Symbiodinium* population of *A*. *millepora* holobiont decreased by 50% when exposed to the future scenario Representative Concentration Pathway, RCP8.5 compared to corals exposed to present day conditions (PD) (Fig 1A, Table 2). This was also reflected in chlorophyll *a* cm^-2^ levels, which were also halved. In concert with these changes, the photosynthetic rates (as measured by P _net_ max cm^-2^ and P _gross_ max cm^-2^) of corals exposed to RCP8.5 future conditions were reduced to 33–41% compared to corals under PD conditions (Fig 1B and 1C, Table 2). Light-enhanced dark respiration (LEDR) was also reduced to 37% as compared to corals under Pre Industrial (PI) conditions (Fig 1C, Table 2). The remaining symbionts in RCP8.5 were less productive (as measured by P _net_ max cell^-1^ and P _gross_ max cell^-1^) in comparison to symbiont populations found in corals exposed to PI scenarios (Fig 1D, Table 2). PI corals were more productive both per symbiont cell (as measured by P _net_ max cell^-1^ and P _gross_ max cell^-1^) and as a whole (as measured by P _net_ max cm^-2^ and P _gross_ max cm^-2^) compared to corals under PD (Fig 1C and 1D).

**Table 1:** Summary of mean seawater temperature and pCO_2_ concentration. Seawater parameters were determined for the 5 week experimental period along with carbonate parameters estimated for experimental treatments, where RCP stands for Representative Concentration Pathway. The five week mean represents the mean of all the parameters over the course of the experiment. Total alkalinity (AT) is a mean of 8 seawater replicate samples per treatment (n = 8) collected weekly both at noon and midnight. Measured parameters are presented with ± standard error of the mean. Output parameters were estimated using the program CO2SYS.

	5- week mean			Output parameters			
Treatment	T (°C)	pCO_2_ (μatm)	A_T_ (μmol kg^-1^)	pH	Ω aragonite	HCO_3_^-^ (μmol kg^-1^)	CO_3_^2-^ (μmol kg^-1^)
Preindustrial (PI)	23.0 ± 0.1	325.4 ± 3.3	2209 ± 1	8.09	3.37	1678	214
Present day (PD)	24.0 ± 0.0	403.6 ± 0.8	2206 ± 2	8.02	3.03	1731	192
RCP 4.5	26.0 ± 0.1	627.5 ± 1.1	2207 ± 1	7.85	2.40	1835	151
RCP 8.5	28.0 ± 0.1	997.7 ± 1.4	2208 ± 1	7.68	1.83	1928	114

**Table 2:** Results of one way ANOVA/Kruskal Wallis test for physiological parameters. These *Acropora millepora* and *Symbiodinium* parameters are presented in Figs 1 and 2, for corals exposed to different temperature and pCO_2_ scenarios (Pre-Industrial, Present Day, RCP4.5 and RCP8.5), where RCP stands for Representative Concentration Pathway.

Physiological parameter in *Symbiodinium* cells/ Coral holobiont	Source of variation	Df	F/H	P
***Symbiodinium***	cell no cm^-2^	3	6.97	< 0.001
	total	42		
	ng chl *a* cell^-1^	3	3.42	0.026
	total	41		
	ng chl *a* cm^-2^	3	11.25	0.010
	total	42		
	ng chl *c*_*2*_ cell^-1^	3	4.61	0.007
	total	40		
	ng peridinin cell^-1^	3	3.91	0.015
	total	41		
	ng β carotene cell^-1^	3	5.35	0.004
	total	41		
	ng (Dtn + Ddn) cell^-1^	3	1.91	ns
	total	40		
	Dtn/ Dtn + Ddn	3	3.66	0.024
	total	40		
	P_gross_ max p moles O_2_ h^-1^ cell^-1^	3	4.03	0.015
	total	40		
	P_gross_ max p moles O_2_ h^-1^ cm^-2^	3	19.89	< 0.001
	total	40		
	P_net_ max p moles O_2_ h^-1^ cell^-1^	3	12.25	0.007
	total	40		
	P_net_ max p moles O_2_ h^-1^ cm^-2^	3	23.58	< 0.001
	total	40		
**Coral holobiont**	LEDR p moles O_2_ h^-1^ cell^-1^	3	2.07	ns
	total	40		
	LEDR p moles O_2_ h^-1^ cm^-2^	3	5.26	0.004
	total	42		
	DR p moles O_2_ h^-1^ cm^-2^	3	17.56	< 0.001
	total	42		
	% change in weight (g)	3	3.13	0.037
	total	42		
	mg water soluble protein cm^-2^	3	5.10	0.004
	total	42		
	mg total lipid cm^-2^	3	12.55	< 0.001
	total	42		

**Fig 2:**
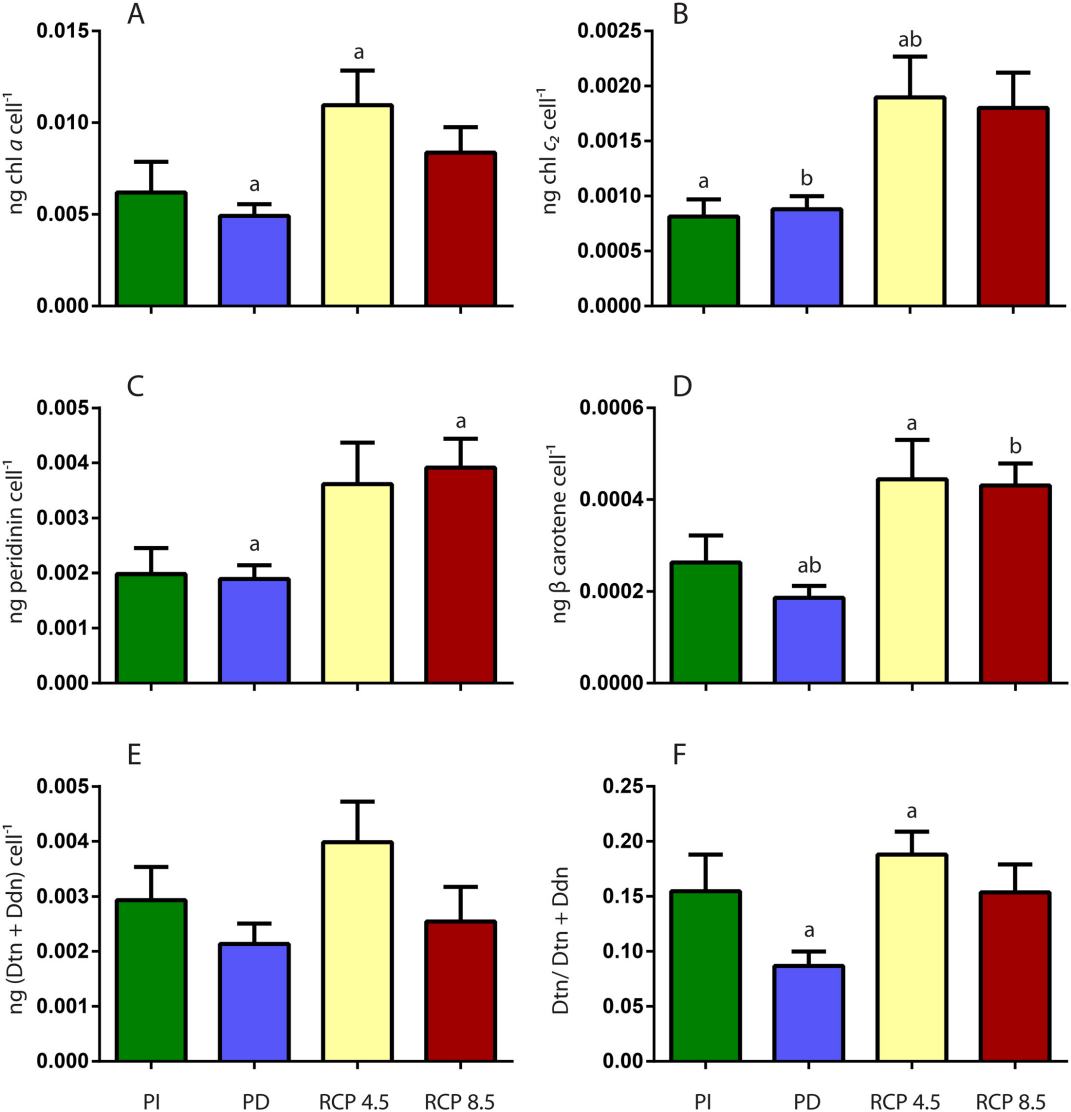
The effect of past and future changes in seawater temperature and pCO_2_ (Pre Industrial, PI, Present Day, PD, Representative Concentration Pathway RCP4.5 and RCP8.5) conditions on *Symbiodinium* pigmentation. (A) Chlorophyll *a* content per cell (B) Chlorophyll *c*_*2*_ content per cell(C) Peridinin content per cell (D) β carotene content per cell (E) Xanthophyll pool (diatoxanthin, Dtn and diadinoxanthin, Ddn) per cell and (F) Xanthophyll de-epoxydation. Error bars represent the standard error of the mean (n = 16). Post hoc differences are provided above the relevant data in letter symbols.

We also report a 2-fold decrease in dark respiration per coral surface area (cm^-2^) and a 75% reduction in calcification/growth (as measured by buoyant weight) for RCP8.5 corals compared to PD corals (Fig 1E and 1F, Table 2). Interestingly there was a 2-fold increase in total lipid content per cm^-2^ for the coral holobiont in RCP4.5 and 8.5 scenarios as compared to PI and PD conditions, and total water soluble content per cm^-2^ increased by 43% in RCP4.5 conditions as compared to the other scenarios (Fig 1G and 1H, Table 2). Photosynthetic pigments in *Symbiodinium* fluctuated between the 4 different scenarios. Chlorophyll *a* cell^-1^ content had a 2-fold increase under RCP4.5 conditions, while Chlorophyll c_2_ cell^-1^ amounts increased by 2-fold in both the RCP4.5 and 8.5 treatments (Fig 2A and 2B, Table 2). Both peridinin and β carotene content were 2-fold higher in RCP8.5 corals as compared to present day corals, while only β carotene was 2-fold higher in RCP4.5 corals (Fig 2C and 2D, Table 2). There was no difference in xanthophyll pool (Diatoxanthin and Diadinoxanthin) content among treatments, while RCP4.5 corals had a 2-fold increase in xanthophyll de-epoxidation (Fig 2E and 2F, Table 2).

### Differentially-expressed genes

Our investigation also explored whether there were broad transcriptomic changes by the *A*. *millepora* holobiont in response to past, present and predicted future changes in both temperature and pCO_2_. RNA-Seq produced 33–62 million reads (99bp) per treatment. The application of the Differential K-mer Analysis Pipeline (DiffKAP) and k-mer analyses (16-mer size) resulted in over 300 000 differentially expressed reads (DERs) per treatment /control comparison. Only around 10% of these DERs were annotated with a significant translated nucleotide query-protein database (BLASTx) alignment (E-value ≤ 10^−15^) to the Swiss-Prot database, which we then equated with differentially-expressed genes (DEGs). With each climate change scenario being compared to PD conditions, there was differential gene expression in 2592 (1032 up and 1560 down regulated) DEGs in the Pre-Industrial (PI) scenario, 2677 (2138 up and 539 down regulated) DEGs in RCP4.5 corals and 1642 (899 up and 743 down regulated) DEGs in RCP8.5 corals (S1 Fig). This represented an overall modification of the metatranscriptome, based on short reads with a significant BLASTx hit to the Swiss-Prot database, by 4.9, 4.6 and 3.1% in PI, RCP4.5 and RCP8.5 corals respectively, compared to PD conditions. There were 210 DEGs which overlapped across all 3 scenarios (S1 Fig). The taxonomic composition of our metatranscriptomes was fairly uniform across treatments, whereby a large proportion of reads were of coral origin (73–79%), followed by reads from *Symbiodinium* (10–13%) and reads from other organisms, including bacteria, viruses and fungi, which represented a small proportion of the total transcripts (~ 1%). There was also a proportion of our reads that did not match any of the taxonomic references (10–15%) (S2 Fig). The highest proportion of *Symbiodinium* DEGs was found in PI compared to PD conditions, where 72% of the down regulated DEGs in the metatranscriptome were of *Symbiodinium* origin. RCP4.5 conditions on the other hand had the highest proportion of DEGs (termed other) that did not match any of the taxonomic references or were of bacterial/viral origin, these DEGs represented 21% of up regulated DEGs in this treatment. Overall, coral DEGs were dominant and represented between 46–83% of either down or up regulated DEGs, except for the PI down regulated DEGs (S1 Table).

We used qPCR analysis of 13 randomly selected gene candidates (S2 Table) based on a gradient of expression from highly up regulated to highly down regulated DEGs. Our results show that there was a correlation between the qPCR and RNA-seq results, based on log_2_ fold gene expression (relative to the control) (r^2^ = 0.79; p<0.0001) (S3 Fig), validating our approach of using the DiffKAP on RNA-seq data.

**Fig 3:**
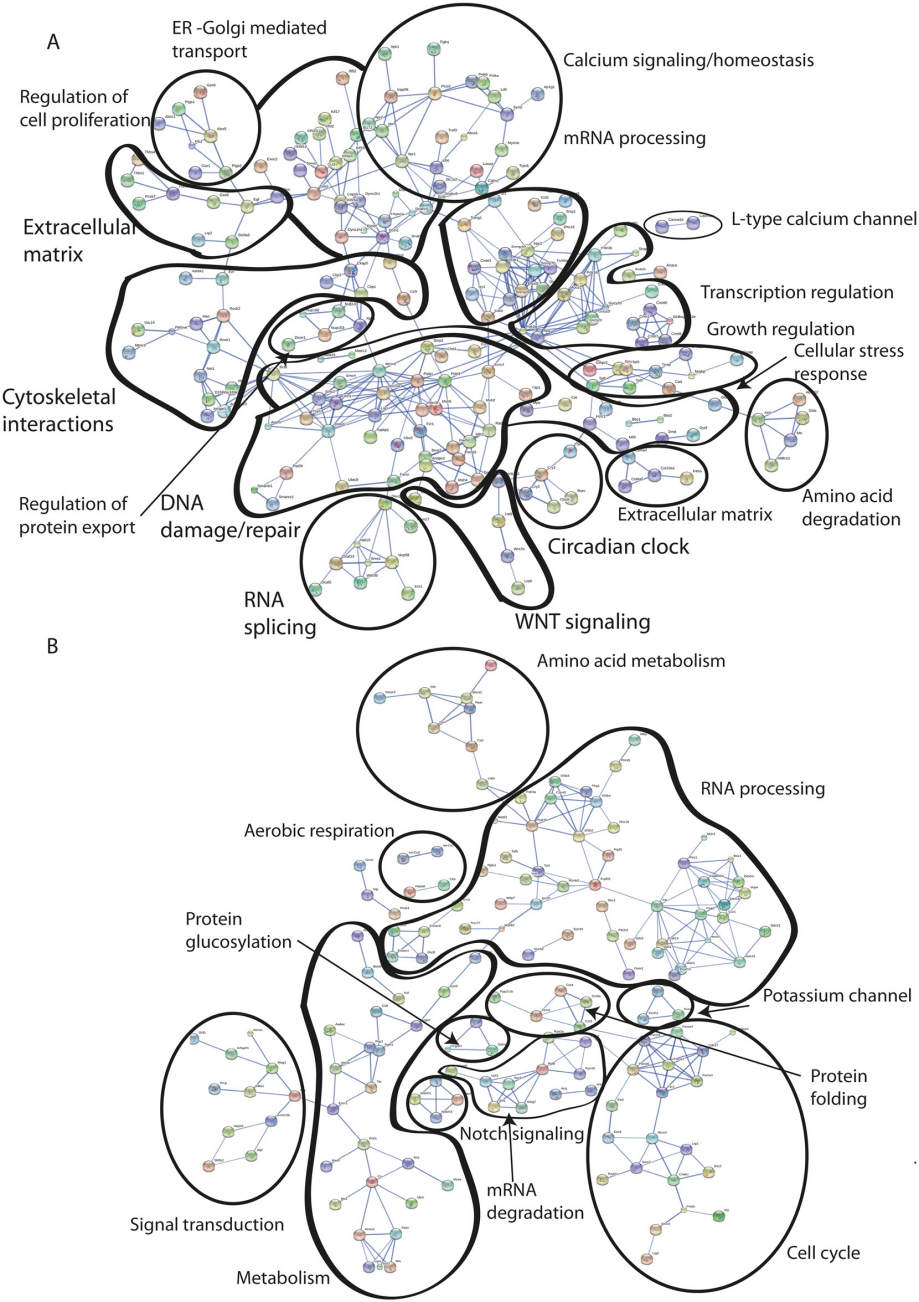
Protein interaction networks. Protein interaction network of genes that were up regulated (A) and down regulated (B) in Representative Concentration Pathway RCP8.5 *Acropora millepora* holobiont. The DEG list of the RCP8.5 vs Present Day (PD) conditions comparison was uploaded to the STRING (Search Tool for the Retrieval of Interacting Genes) database. Protein interactions are categorized according their Gene Ontology classifications.

Overall, the largest increase in transcript abundance was seen for GFP-like non-fluorescent proteins, toxins/cytolysis, genes involved in metabolism and photosynthesis under PI conditions (see S1 Table). Under RCP4.5 conditions many highly expressed ribosomal transcripts were found together with transcripts involved in microtubule based processes and cilium movement. In contrast, under RCP8.5 conditions, we saw an increase in transcripts involved in oxidative stress, calcium signaling/homeostasis, cytoskeletal interactions, transcription regulation, DNA repair, Wnt signaling and apoptosis/immunity/ toxins (including NF-κB signaling) and circadian clock genes (Fig 3, S1 and S3 Tables). There was a decrease in transcripts for pyruvate metabolic process, small GTPase mediated signal transduction and microtubule based processes under PI conditions. RCP4.5 coral holobionts had down regulation in Carbonic anhydrase, fatty acid biosynthesis and transcripts involved in transcriptional regulation. Under RCP8.5 conditions, there was a down regulation in transcripts involved in metabolism, RNA processing, cell cycle, fatty acid biosynthesis, glucose transporters, carbonic anhydrases, bicarbonate transport, skeletal organic matrix proteins and the Notch signaling pathway (Fig 3, S1 and S3 Tables).

### Gene ontology enrichment analysis and protein interactions

**Fig 4:**
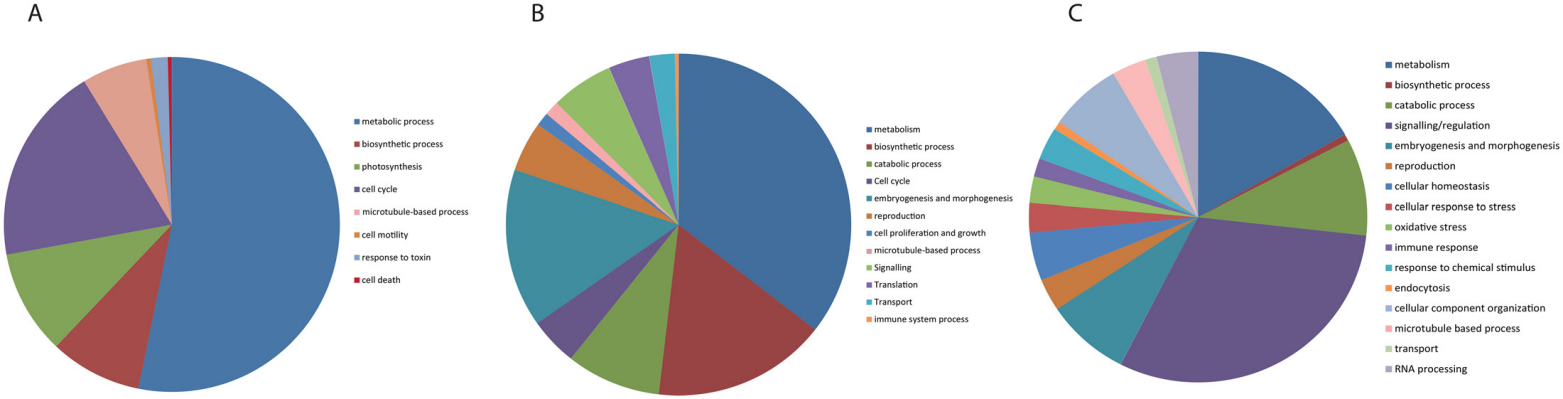
Gene enrichment analysis. Gene enrichment analysis (P<0.05) of biological processes for differentially expressed genes in *Acropora millepora* holobiont exposed to Pre Industrial (PI) vs Present Day (PD) conditions (A) Representative Concentration Pathway RCP4.5 vs PD conditions (B) and RCP8.5 vs PD conditions (C). The program DAVID was used to test for enriched GO categories among differentially expressed genes. Each pie segment is annotated for specific GO category and the sizes of the pie segments are proportional to the total number of genes enriched.

Global gene expression variations within the metatranscriptomes across temperature and pCO_2_ scenarios were further explored through GO enrichment analysis. When comparing corals from PI to PD conditions, there were 39 biological processes and 56 molecular functions enriched (S1 File), which when grouped into mother GO categories (Fig 4A) they showed that changes in metabolic processes in the coral holobiont are dominant under the PI scenario. Metabolic processes were dominant under all 3 scenarios; however, the proportion of enrichment was the largest under PI conditions. Also, the microtubule-based process category was the largest in PI corals among the 3 treatments. In addition, we saw changes in photosynthesis, cell motility, response to toxin and cell death and these changes were not present under RCP4.5 and RCP8.5 conditions (Fig 4A). RCP4.5 corals had 189 biological processes and 55 molecular functions enriched (S1 File), and showed that a large proportion of biological processes enriched were involved in biosynthetic process, catabolic process, embryogenesis and morphogenesis, and reproduction (Fig 4B). Both PI and RCP4.5 corals had enrichment in cell cycle processes, but the proportion of enrichment was larger under PI conditions. In addition, embryogenesis and morphogenesis, and reproduction processes which were also enriched under RCP8.5 conditions showed a larger proportion of enrichment under RCP4.5. Unique GO categories enriched under the RCP4.5 scenario also included translation and cell proliferation and growth (Fig 4). Corals exposed to the future scenario without emission reductions (RCP8.5), showed GO enrichment for 138 biological processes and 49 molecular functions (S1 File). Under this scenario, the metabolic processes proportion was the smallest among the 3 past and future temperature and pCO_2_ conditions and signaling/regulation formed the largest proportion of enrichment (Fig 4C). GO categories only found under RCP8.5 included cellular homeostasis, cellular response to stress, oxidative stress, response to chemical stimulus, endocytosis and RNA processing. The biosynthetic process, embryogenesis and morphogenesis, and reproduction categories formed a much smaller proportion of the enrichment, while immune response was a larger proportion as compared to corals under RCP4.5 conditions. There were 210 DEGs which overlapped across all treatments (S1 Fig), and GO enrichment analysis of these showed that the biggest proportion of these categories were biological processes involved in microtubule-based movement, cellular component organization and assembly, and multicellular organismal development (S2 File). Significantly enriched pathways were found in RCP4.5 corals which had a number of genes found in the ribosome, while RCP8.5 corals had enriched ubiquitin-mediated proteolysis pathways (Table 3).

**Table 3:** KEGG pathway analysis. Gene enrichment analysis showing KEGG pathways involved in thermal and ocean acidification stress for *Acropora millepora* exposed to reduced emissions Representative Concentration Pathway (RCP4.5) and business as usual (RCP8.5) scenarios.

Pathway	No of genes	No of KEGG genes in pathway	Fold Enrichment	Corrected P-value
**RCP4.5**				
Ribosome	22	122	8.2	8.37E-11
**RCP8.5**				
Ubiquitin mediated proteolysis	14	143	7.2	4.13E-05

In order to gain further understanding of the cellular processes occurring during coral holobiont stress, as seen at the phenotypic level (Fig 1), we investigated the interactions among proteins encoded by up and down regulated genes in the coral holobiont, under RCP8.5 conditions (Fig 3). Up regulated DEGs point to a large group of proteins involved in sensing and repairing DNA damage, which is connected to cellular stress response, WNT signaling, circadian clock control, cytoskeletal interactions, mRNA processing and transcription regulation. The cellular stress response cluster was connected to an extracellular matrix group consisting of several collagen types. A group of growth-regulating proteins, responsible for regulating cell-cycle arrest, was connected to the transcription regulation cluster. Another group of up regulated proteins involved with calcium signaling/homeostasis was linked with ER-Golgi-mediated transport, which in turn was linked to control of cell proliferation and organization of the extracellular matrix (Fig 3A). Proteins involved in the ubiquitin-mediated proteolysis (Table 3) were interacting with proteins involved in DNA repair, calcium/signaling homeostasis, cellular stress response and ER-Golgi-mediated transport (Fig 3A). Down regulated proteins included large clusters of metabolism, cell proliferation, RNA processing and cell cycle. Here the metabolic cluster was connected to the down regulation of cell proliferation and RNA processing. Cell cycle down regulation is connected to protein folding. Among down regulated clusters we also saw members of the Notch signaling pathway and proteins involved in aerobic respiration (Fig 3B).

## Discussion

### Changes in selected physiological processes of the phenotype

Our study shows that exposure of the reef-building coral, *A*. *millepora*, to predicted future pH and temperature conditions under the business-as-usual projection RCP8.5, results in corals with reduced *Symbiodinium* populations and chlorophyll *a* levels, indicating mild coral bleaching, these results support previous findings on the compounding effect of the interaction between increased temperature and OA stress, and where destabilization of the coral-algal symbiosis occurs at lower thermal thresholds [19,20,35]. We also report major changes in photosynthesis and respiration, in addition to decreased calcification (Fig 1). Although our study cannot shed light on the adaptation or acclimatization potential of reef building corals to future ocean temperature and acidification conditions, we provide information about potential impacts for branching corals under such conditions. Our evidence for decreasing rates of gross photosynthesis per surface area and *Symbiodinium* cell, compounded by reduced *Symbiodinium* populations, indicates disruption of photophysiological processes in *Symbiodinium* and may lead to a reduction in photoassimilates translocated to the host coral. Such results can have serious impacts on the ability of the host to recovery from bleaching. Of considerable interest, was the observation that there was a 2-fold downturn in dark respiration per coral surface area (cm^-2^) which suggested reduced growth rates and/or metabolism under these environmental conditions. These changes are likely to have long-term negative effects on host growth and fecundity, with the prospect of increased susceptibility to disease and mortality, especially if *Symbiodinium* populations fail to recover rapidly [36]. Interestingly, there was an increase in photosynthesis for PI corals compared to their conspecifics under PD conditions (Fig 1C and 1D), which perhaps reflects that these corals are more suited to ocean conditions seen 100 years ago, and is consistent with mesocosm studies finding reef communities performing better under pre-industrial conditions [24]. In our study, however, increased productivity in PI corals did not translate into higher tissue growth or calcification rates. This may be a consequence of the short time frame of the experiment (5 weeks) and it is possible that this benefit may have been observed if a longer experimental incubation was carried out. Regardless, implications of these results need to be taken into account when speculating on the potential of reef builders to acclimatize or acclimate to future conditions (as stressed by [24]). Despite variability among reef calcifiers, overall calcification rates have been predicted to decrease by the end of the century, as a result of changes in both pCO_2_ and temperature [4,37–39]. Our results show that corals exposed to conditions like that of RCP8.5 had negative calcification rates and further emphasize the extent of challenges that reef building corals are likely to face in warmer and more acidic future oceans. Corals have the ability to maintain cell pH homeostasis, as it is critical to a range of cellular functions [40] and can up-regulate the pH at the calcification site, even under highly acidified conditions [41–43]. These pH regulation processes require elevated maintenance costs [4,22,23,44]. In our study, under both higher pCO_2_ and temperature, our results do not reflect a decline in energy reserves (total lipids and proteins); in fact, we saw an increase in lipids for both RCP4.5 and RCP8.5 corals, and an increase in proteins under RCP4.5 conditions (Fig 1). However, it is important to note that our measure of total lipids did not separate the coral fraction from the *Symbiodinium* fraction, and it may be that *Symbiodinium* plays an important role in determining lipid levels in *Acropora millepora* under different environmental conditions, as it has been shown in other coral species [45]. Our results showing an increase in lipids fit well with observations that under long term exposure to OA, some coral species are able to survive despite losing their skeleton and changing into large solitary polyps with three times the biomass of coral colonies under controlled conditions [46]. Although we cannot relate our results to changes in acidification alone, changes observed in the present study also correlate well with changes in energy reserves for *A*. *millepora* [47], where energy reserves were not metabolized as a function of increased acidification or increases in both temperature and acidification. Schoepf et al [47] had similar results in that calcification declined under high OA and temperature, while lipid concentrations increased. It was suggested that maintenance of energy reserves such as lipid concentrations under environmentally stressful conditions may enable corals to maintain their reproductive output [48]. This may explain the results in our study, as our *A*. *millepora* corals were exposed to the experimental conditions just before their annual spawning event.

Given that exposure to increased temperatures can lead to photosynthetic dysfunction in *Symbiodinium* [36] and that inhibited photosynthesis in can influence sensitivity to OA and host cell recovery from cellular acidosis [49], our exploration of *Symbiodinium* pigment profiles in addition to measuring photosynthesis provided further insight into the photosynthetic capacity of the coral holobiont. Corals can adjust photosynthetic pigment concentrations to maximize irradiance levels available for the photosynthetic endosymbiont [50]. Despite a reduction of the endosymbiont population in RCP8.5 corals, the chlorophyll *a* level per cell did not change in comparison to PD and PI corals, while in RCP4.5 corals chlorophyll *a* concentrations per cell increased (Fig 2). In addition, RCP4.5 corals also increased their concentrations of accessory photosynthetic pigments per cell (Fig 2). This may reflect the ability of RCP4.5 corals to adjust their photosynthetic unit under changed environmental conditions, to maximize light utilization and potentially higher productivity, which was also observed at the per cell level in RCP4.5 corals (Fig 1D). RCP8.5 corals did increase accessory pigments levels, which in this case may reflect the changed light environment within these corals as the endosymbiont population was reduced by half and accessory pigments were needed to absorb excess light energy [51]. High light exposure has been also been shown to induce carotenoid production in corals [52]. Xanthophyll de-epoxidation, *i*.*e*. the conversion of diadinoxanthin (Ddn) to diatoxanthin (Dtn) upon light absorption, can alleviate high light stress through dissipation of absorbed radiation to heat [53–55]. Only RCP4.5 corals had greater de-epoxidation (Dtn/Dtn+Ddn) compared to PD corals. A correlation between an increase in de-epoxidation and greater non photochemical quenching has been shown [56]. This is consistent with a possible greater need for non-photochemical quenching in RCP4.5 corals under changed environmental conditions [57]. The observation that RCP8.5 corals did not change their de-epoxidation levels compared to PD corals may reflect that photosynthesis is not functioning adequately in these coral-algal complexes, supporting the downturn in productivity (Fig 1C and 1D) and metabolic suppression (Fig 1E). Energy dissipation is essential for organisms exposed to environmental stress, where an increased degree of excessive light absorption is accompanied by an increased need for energy dissipation [57]. The lack of an up-regulation of xanthophyll de-epoxidation in this case, may point to potential oxidative stress and/or damage present within the coral holobiont.

### Global gene expression response: Pre-industrial scenario

Our analysis of the global metatranscriptome changes in *A*. *millepora* allowed us to identify potential molecular pathways and candidate genes involved in the holobiont response of selected physiological processes seen at the phenotype level (Figs 1 and 2), and to predict changes during past and future ocean warming and chemistry conditions. Although the coral holobiont is composed of the coral host, *Symbiodinium* populations and other symbiont partners (bacteria, viruses, fungi and others) [58–60] our global gene expression response was dominated by coral and secondly *Symbiodinium* genes, while genes from the microbial community in the coral holobiont were a small proportion. This limits our study’s ability to determine important changes that may be occurring in the coral holobiont microbial partners which may have affected the changes that we saw in physiological processes at the phenotype level. We also did not measure changes in microbial communities in our coral branches under experimental treatments and acknowledge the importance of measuring fluxes in microbial communities in future studies to explore what role they may play. Over half of the modifications to the metatranscriptome in corals exposed to PI temperature and pCO_2_ conditions were involved in metabolic processes, such as generation of precursor metabolites and energy, and protein metabolic processes (Fig 4A, S1 Table). Our pattern of coral host metabolic transcripts involved in generation of precursor energy pathways such as glycolysis, being up regulated while metabolic transcripts for glycolysis, Calvin cycle and photorespiration of *Symbiodinium* origin were down regulated (S1 Table) implies a complex array of metabolic pathways in host and endosymbiont underpinning an increase in photosynthesis seen at the phenotype level and warrants further investigation to understand the underlying mechanisms. Photosynthesis was also a major target of transcriptional modulation, where *Symbiodinium* transcripts for psbA, psbB, psbC, psbD1 and petB were up-regulated (Fig 4A, S1 Table), reflecting increases in photosynthesis seen at the physiological level (Fig 1) and suggesting greater energy production potential for PI corals as compared to their PD conspecifics. Our *Acropora millepora* colonies contained the C3 clade of *Symbiodinium*. In a study that compared the transcriptomes of four Symbiodinium clades (A, B, C and D) [61], found that photosynthesis related transcripts were highly conserved and shared among all four clades and therefore supporting the notion that they are unique for dinoflagellates and have not changed through evolutionary time. The observed increase in cell cycle processes, including mitotic cell cycle, and DNA duplex unwinding during replication and M phase (Fig 4, S1 File), may reflect an increase in growth either in the endosymbiont population and/or coral host. In addition, the fact that PI conditions resulted in the largest transcriptional regulation of *Symbiodinium* genes further points to changes in the endosymbiont population. These changes in metabolism, photosynthesis and cell cycle processes seen at the transcriptional level, reiterate the fact that past environmental conditions may in fact be more beneficial for *A*. *millepora*, perhaps suggesting that adaption to present-day conditions has not yet occurred.

### Global gene expression response: reduced emission scenario (RCP4.5)

In response to environmental perturbations in ocean temperature and chemistry simulated by the emissions scenario (RCP4.5), a large part of the biological function of changes to the metatranscriptome belonged to metabolic and biosynthetic processes (Fig 4B, S1 Table). These findings may point to a greater energy potential for RCP4.5 corals as compared to PD corals, supporting the increase in photosynthesis per algal cell seen at the phenotype level (Fig 1D). This may be due to an increase in temperature and pCO_2_, both of which have, in the past, been shown to increase productivity [20]. Although not seen in this study due to the short time frame, Acroporid corals are likely to be stressed and show signs of bleaching and reduced calcification under an RCP4.5 scenario, especially after longer exposure to these conditions and during and/or after the summer [24]. A range of Ribosomal proteins were highly expressed under RCP4.5 conditions (Table 3, S1 Table) and may suggest that cell growth is occurring in response to environmental change. Ribosome biogenesis regulation is a key element in controlling cell growth as ribosomes are required for growth and also because ribosome biogenesis consumes a large proportion of cellular energy. In fact, in a growing cell, up to 95% of total transcription can be attributed to ribosome biogenesis [62]. This is consistent with gene enrichment in cell proliferation and growth, and cell cycle biological processes (Fig 4B). The increase in reproductive processes as compared to PD corals is likely a reflection of this experiment occurring just prior to the annual spawning event, and RCP4.5 corals being exposed to higher temperatures. It has been shown that when corals are exposed to higher temperatures during the late period of gametogenesis, spawning can occur earlier in the spawning month [63], which may explain why in our study, there was an increase in genes related to reproductive events in RCP4.5 corals.

### Global gene expression response: business as usual scenario (RCP8.5)

#### Metabolism

Under the business as usual scenario, RCP8.5, the *A*. *millepora* holobiont showed signs of a stress response at the phenotype level through reductions in endosymbiont population density and chlorophyll *a* concentrations, reduced productivity and decreased respiration rates that may point to potential metabolic depression (Fig 1). The cellular stress response was also apparent on the global gene expression level. Gene enrichment analysis showed that RCP8.5 corals had the smallest proportion of metabolic biological processes compared to corals exposed to RCP4.5 or PI conditions (Fig 4). This is also supported by an overall down-regulation of transcripts involved in glucose metabolism, TCA and oxidative phosphorylation, indicating reduced oxidative metabolism and capacity to generate ATP and NADPH, as well as a reduction in coral sugar and glucose transporters (Fig 3B, S3 Table). Also, biosynthetic processes were less represented as compared to corals under PI and RCP4.5 conditions, perhaps reflecting a decrease in energy-requiring pathways of metabolism. In addition, there were fewer transcripts dedicated to reproduction, embryogenesis and morphogenesis as compared to RCP4.5 corals, which may again point to a more negative energy balance in stressed RCP8.5 corals. Metabolic depression in this study could also be a result of cellular acidosis as our RCP8.5 corals had decreased photosynthesis and reductions in *Symbiodinium* populations (Fig 1) which may point to photosynthetic inhibition, and it has been shown that *Symbiodinium* photosynthetic activity is tightly coupled to the ability of the host cell to recover from cellular acidosis after high pCO_2_ exposure [49]. It is common for organisms to depress their metabolic rates as a result of environmental stress, to temporarily deal with adverse conditions [64]. Metabolic depression seen in this study, however, is particularly alarming if it occurs during prolonged time frames, as corals are unable to return to their normal resting metabolic rate [22].

#### Circadian clock

The circadian clock is a core mechanism in nearly all organisms which controls and synchronizes physiological processes to the day/night cycle. As such, circadian rhythms are important in controlling the metabolism of an organism (see review, [65]). It has been shown that disruptions to circadian rhythms, whether environmental or genetic, can have profound effects on metabolism and lead to metabolic diseases in mammals [66]. There are components of the circadian clock that can sense alterations to the cell’s metabolism, and it has been suggested that metabolism is not only affected by the circadian clock, but can also act as a modulator of the circadian clock [65]. In corals, like in many organisms, metabolic gene expression is linked to circadian rhythms [67]. Given the dramatic change in metabolism in terms of decreased respiration rates (Fig 1) and down regulation of coral transcripts involved in oxidative metabolism, indicating potential metabolic depression (S3 Table) seen in our RCP8.5 corals, it is interesting to note that elements of the coral circadian clock machinery are up-regulated (Fig 3A, S3 Table), and NPAS3 has been shown to be involved in the circadian regulation of gluconeogenesis in mammals [68,69]. This may suggest that the circadian clock in corals is intrinsically linked, perhaps to controlling metabolic events (such as metabolic depression) during environmental perturbations. This is an avenue that needs to be explored in future studies.

#### Increasing cellular stress

In response to environmental stressors of increased temperature and OA, our measurements showing declines in *Symbiodinium* densities and pigment concentrations indicated a breakdown in the coral-algal symbiosis. Overall, our metatranscriptomic analysis points to a cellular stress response which is a universally conserved mechanism for protecting macromolecules within cells from potential damage resulting from abiotic stress [70]. It has been suggested that in *Stylophora pistillata* exposed to heat stress, the cellular stress response is to engage in Endoplasmic Reticulum (ER)-unfolded protein response (UPR) and ER associated degradation (ERAD) [33]. On the other hand, analysis of global gene expression in adult *Pocillopora damicornis* exposed to extreme CO_2_-driven pH declines of ≤ 7.4, showed a lack of cellular stress and changes were seen in transcripts associated with the transport of calcium and carbonate ions, organic matrix, photosynthesis and increased energy metabolism [29]. Our adult corals, which were exposed to both an increase in temperature and a CO_2_-driven decrease in seawater pH, did show a cellular stress response which was similar to that seen in heat-stressed *S*. *pistillata* [33], perhaps supporting the notion that exposure to OA can lower the thermal threshold for corals to experience thermal stress [19,20,35]. Our results point to up-regulation of processes involved in oxidative stress, DNA repair, Ubiquitin-mediated proteolysis, apoptosis and toxins (S1 File, Fig 3, S3 Table). We also saw modulations in calcium signaling/homeostasis and cytoskeletal interactions (Fig 3, S3 Table), which have previously been shown to be part of the coral holobiont stress response [23,30,33]. Nuclear factor (NF)-κB signaling can regulate physiological processes involved in innate immune response, cell death and inflammation (see review [71]). The co-expression of transcripts involved in NF-κB signaling (*e*.*g*. Traf3) with transcripts encoding for toxins and cytolysis, suggests that this network of genes may be involved in innate immunity in our stressed coral holobionts. Stress-induced breakdown of the coral-algal association can result in shifts in microbial communities and increased susceptibility to coral pathogens [72,73]. This may be why there is an increase in pathways potentially involved in innate immunity in the stressed RCP8.5 corals. There were 14 genes enriched in the Ubiquitin-mediated proteolysis pathway (Table 3, S3 Table), which is involved in protein degradation and turnover [74]. Eight of the Ubiquitin-mediated proteolysis enriched transcripts found in this study (UBLE1B, UBE2D_E, UBE2J1, UBE2W, HERC2, CYC4, F-box and Apc2), were the same as what Maor-Landow et al [33] found in their heat-stressed corals. It has been suggested that protein ubiquitination and an increase in ubiquitin expression levels is a sign of cellular heat stress [75]. Potential protein degradation found in this study may be one of the cellular markers for both temperature and ocean acidification stress in corals. We also observed a down-regulation in Notch signaling, which was also seen in heat-stressed *S*. *pistillata* [33]. The observation of up-regulation in a range of coral and *Symbiodinium* transcripts encoding oxidative stress DNA damage/repair, molecular chaperones, ER stress and apoptosis proteins under RCP8.5 conditions (S3 Table), further point to a cellular stress response [23,30,33]. These changes in the adult coral gene expression profiles also further supports the notion that the coral stress response is fairly similar across a range of environmental stressors such as temperature [30–32], darkness [76] and low pH [23].

#### Modification of calcification

Many genes involved in calcification in corals are likely to be taxonomically restricted and unique to corals [28]. As our transcriptomic results only refer to transcripts with hits to known proteins, we are unable to compare potential genes involved in the calcification process [28] which have no hits to the Swissprot database. At the phenotype level, we observed a decrease in biomineralization (Fig 1) under the RCP8.5 scenario. Interestingly, we saw an increase in LRP5 and other components of the canonical Wnt signaling pathway (Fig 3A). In human skeletons, LRP5 signaling has been shown to regulate bone mass [77], and it may be that in corals, this pathway can play a role in calcification, which could be an avenue for future work. Global gene expression patterns showed a complex pattern of up-regulation of coral T-type calcium channel and up-regulation of other calcium transporters, while there was a down-regulation in bicarbonate transport, carbonic anhydrases and transcripts potentially implicated in the organic matrix (S3 Table). T-type calcium channels have been suggested to be associated with bone development in vertebrates [78,79], and the fact that up-regulation of these was found in RCP8.5 corals in this study, and in the high CO_2_ treatment in [28], may imply that these calcium transporters are part of the coral response to acidification stress, although further studies should elucidate their exact roles. Up-regulation of transcripts for a voltage-gated Ca^2+^ channel was also found in *P*. *damicornis* adult corals in response to ocean acidification conditions [29]. In our study, we had an up-regulation of Ca^2+^ channels of coral origin, other than those found in [28], suggesting that an increase in both temperature and pCO_2_, as manipulated in this study, has either a direct effect (transport of calcium to the extracellular medium) and/or an indirect effect on calcification, through changes in central calcium signaling pathways (Fig 3). Carbonic anhydrases catalyze the inter conversion of HCO_3_^-^ and CO_2_, and are involved in pH regulation. In corals, they are also involved in biomineralization and carbon exchange between host and endosymbiont [80–82]. Similar to results seen in [28], we report down-regulation for one bicarbonate transporter (SLC4a4), and down-regulation of six coral carbonic anhydrases; while there was up-regulation of one *Symbiodinium* carbonic anhydrase, which differs to results seen in [29] where an up-regulation of several bicarbonate transporters and two carbonic anhydrases was seen under acidification levels comparable with our study. The differences between our results and those seen in [29] are likely to be due to difference in coral species and experimental conditions used, especially highlighting that our study measured the effect of combined temperature and OA stress. Our study also highlights a down-regulation in a few coral transcripts (Bmp6 and SVEP1) that may encode for skeletal organic matrix proteins. In our study, modulations to ion transport and potential matrix proteins support our findings at the phenotype level, where there was a reduction in biomineralization—a change that was not measured in both [28,29], which highlights the necessity to measure phenotypic alterations so that modulations at the global transcriptomic level can help us understand the molecular processes underlying the phenotypic change.

#### Cell-wide responses to RCP8.5

**Fig 5:**
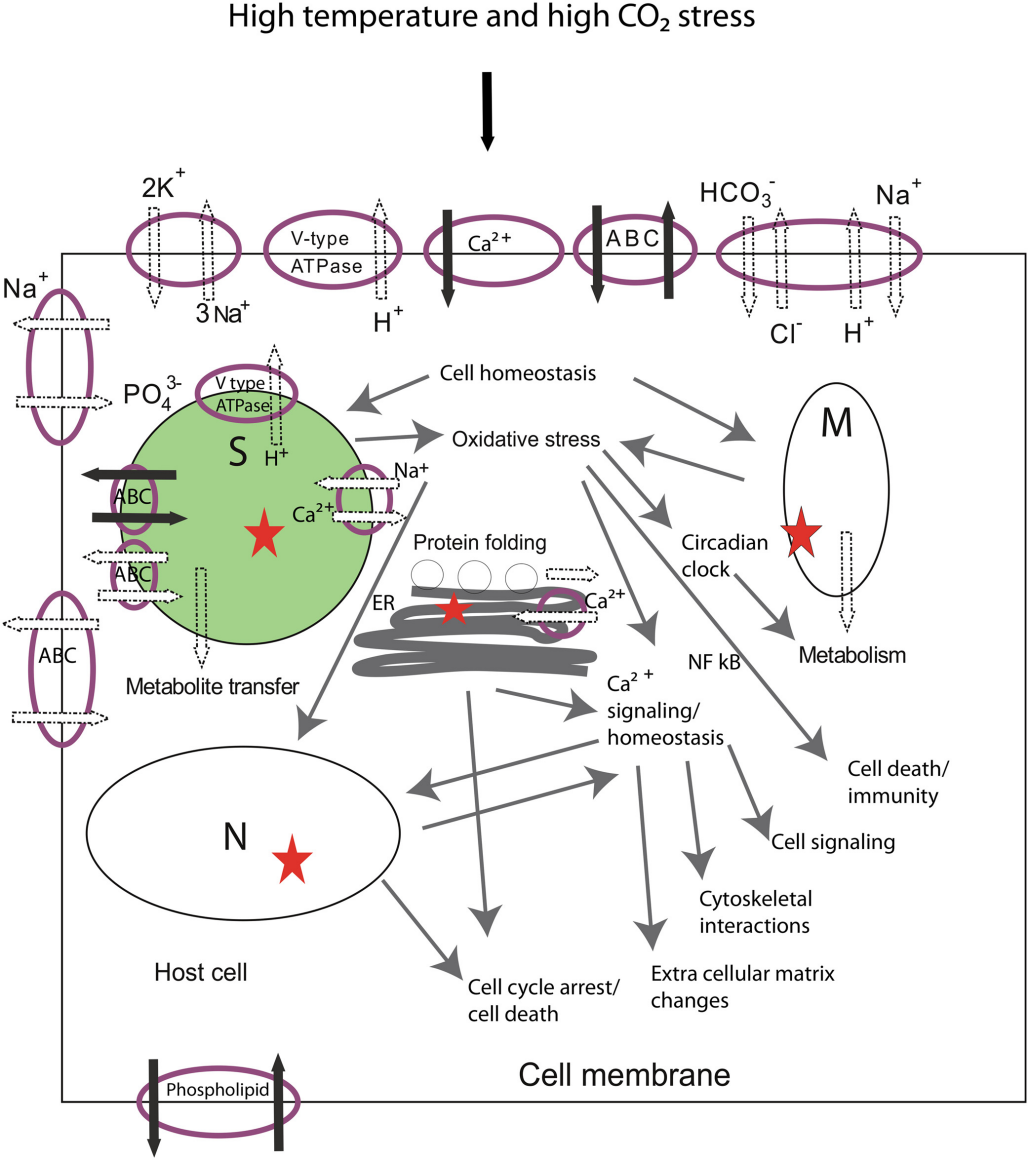
A proposed model of cellular events occurring in response to future Representative Concentration Pathway RCP8.5 scenario with increased temperature and high CO_2_ conditions (for gene list see S3 Table). These changes lead to compromised health in *Acropora millepora* (reduction in symbiont cells, decreased photosynthesis and respiration and reduced calcification). The schematic depicts an endodermal cell which contains the *Symbiodinium* cell. Cellular events depicted here, especially acid base regulation at the cell membrane are also likely to occur in other cell types which do not contain symbiont cells. Exposure to increased temperature and seawater carbonate chemistry leads to changes in acid base regulation and ion transport at the cell membrane both in coral host and in the symbiont cell. Despite regulatory changes at the cell membrane cellular homeostasis may not be reached and acidosis may occur. The result may be an increase in reactive oxygen species due to a disruption (*) in the symbiont cell (S) and /or in the coral host mitochondrion (M), which may also produce reactive nitrogen species. In addition DNA damage may increase and not equal repair mechanisms in the cell nucleus (N) and / or M and S, which can lead to cell cycle arrest and /or cell death. Oxidative stress and endoplasmic reticulum (ER) stress, due to mis-folded protein accumulation and increased ubiquitin mediated proteolysis, may also lead to cell cycle arrest and eventually cell death, in addition to affecting cell calcium homeostasis/signaling, which in turn may lead to modifications in the extracellular matrix, cytoskeletal interactions and cell signaling. Oxidative stress can also affect immunity and /or cell death through the NF-κB pathway. In addition to the disruption in both S and M leading to suppression of metabolism, the coral circadian clock can also be influenced by oxidative stress and/or change in cell metabolism and in turn affect coral host metabolic pathways, which in this case is metabolic depression. Black arrows indicate up regulation and white arrows indicate down regulation.

Based on phenotype-level changes and global gene expression levels, we propose a model of cellular events occurring in the *A*. *millepora* holobiont in response to future ocean temperature and chemistry conditions as predicted by the RCP8.5 scenario (Fig 5). This is an attempt to highlight how the proposed coral heat stress mechanisms of URP and ERAD [30,33] may fit with the coral specific acidosis response in terms of acid base regulation [23,28,29], metabolic depression [23,28], and novel responses of DNA damage/repair and circadian clock modifications observed in this study. Evidence of acid base regulation changes and ion transport in both coral host and endosymbiont (Fig 3, S3 Table) indicate that cellular homeostasis may not have been reached under new environmental conditions, as proton transport out of the cell is overall down-regulated both by the host and as a result of decreased photosynthesis [49]. As a result, damage or disruption to either endosymbiont cell and/or both host cell/ mitochondrion and nucleus may occur. The result is an increase in oxidative stress and DNA damage, as indicated by an increase in oxidative stress and DNA damage/repair transcripts (Fig 3, S3 Table). This can, in turn, lead to changes in calcium signaling/homeostasis [23,32,36], which has an effect on modifications in the extracellular matrix, cytoskeletal interactions, cell signaling (including transcription regulation and RNA processing) and immunity/cell death through the NF-κB pathway (Fig 3A, S3 Table). Additionally, evidence of up-regulation of the ubiquitin-mediated proteolysis pathway and ER stress (Fig 3, Table 3, S3 Table) suggest that cellular energy may be used for the UPR response [30,33], and this may lead to cell cycle arrest and control cell proliferation/death. The DNA damage/repair balance may also regulate cell proliferation and lead to cell cycle arrest, as well as regulate other signaling events [83] (Fig 3, S3 Table). Disruption in endosymbiont and/or host can also lead to metabolic suppression as seen by down-regulation of metabolic transcripts involved in energy metabolism in both the coral host and *Symbiodinium* (Fig 3, S3 Table), and evidence at the phenotype level (Fig 1). The up-regulation of circadian clock components (Fig 3A, S3 Table) may point to a role that the coral clock can play in regulating metabolic pathways in a stressed coral host (in this case, suppressing metabolism). Since, in corals, the genes involved in stress response and cell protection are tightly linked to the circadian clock [67], we suggest here that in our stressed coral, the increase in oxidative stress and changes in metabolic transcripts create a feedback loop in which the circadian clock genes are modulated, which in turn affects coral metabolic pathways (Fig 5).

### Conclusions

Our results at both the phenotypic and global gene expression level indicate that Acroporid corals are highly sensitive to predicted future increase in ocean temperature and OA, and may fail to thrive and show decreased calcification. This phenotypic response is perhaps not a direct result of reduced calcification as this process can be sustained at high pCO_2_ levels [29,41,43], but instead a by-product of increased maintenance cost under new environmental conditions of high temperature and pCO_2_, and diversion of cellular ATP to pH homeostasis, oxidative stress response, UPR and DNA repair. This, together with metabolic suppression, can result in decreased amounts of energy available for calcification and other cellular functions, which may in the longer term lead to increased levels of coral mortality. In order to elucidate mechanisms for the large intraspecific variability of coral sensitivity to both thermal and ocean acidification stress, there is a need for future long-term studies of a range of coral species exposed to modulations in temperature and pCO_2_ both in isolation and as a combined effect. In addition there is a need for studies to investigate the adaptation or acclimatization potential of reef building corals to future ocean temperature and acidification conditions.

## Materials and Methods

### Experimental design

A total of 400 branches (6cm long) were collected from 16 healthy (displaying fully pigmented tissues) colonies of the reef-building coral, *Acropora millepora*, on Heron Island reef flat (23 33’S, 151 54’E), Great Barrier Reef, Australia (in accordance with Great Barrier Reef Marine Park Authority permit G09/32853.1). Coral branches were affixed to cut 15 mL falcon tubes using Selleys Knead It Aqua (Padstow, Australia) and Selleys autofix super glue (Padstow, Australia). The affixed branches were then placed onto a rack which was deployed back to the Heron Island reef flat, where they remained for 6 weeks, exposed to natural light and flow regimes in order to recover from handling. Based on previous experiments on this coral species and location we have found that 6 weeks is sufficient time for branches to recover from handling based on observations of tissue regrowth on affixed surface, dark pigmentation of coral branch and healthy chlorophyll *a* fluorescence measurements determined by a pulse amplitude modulation fluorometer (Diving-PAM, Walz, Germany). Following this acclimatization period, the coral branches were transferred to aquaria with flowing seawater, ambient light (with neutral density filters; see below) and ambient temperature (24°C) conditions for 10 days, as flow through sea water in aquaria is taken straight from the reef flat where the colonies were collected and subsequently acclimatized for 6 weeks. During the 10 acclimation period in aquaria chlorophyll *a* fluorescence measurements recorded by a Diving-PAM, and healthy values of dark adapted yields of ~ 0.7 were observed after this period across all aquaria, and tissue pigmentation did not change. The treatment conditions were then slowly increased in the aquaria over the course of 5 days (rate of change was 0.5–1°C /day). For each treatment, there were four randomly distributed aquaria and for each *A*. *millepora* colony, branches were evenly distributed across treatments (all 16 colonies were exposed to all 4 treatments), with 6 branches per colony in each of the treatment tanks (Pre Industrial, Present Day, RCP4.5 and RCP8.5: Table 1), and in each of the 4 replicate treatment tanks there were branches from 4 out of the 16 *A*. *millepora* colonies. The duration of the experiment was 5 weeks (Oct 4^th^–Nov 8^th^ 2010) and coral branches were sampled at time zero (after the 10 day of acclimation period before experimental exposure) and after 5 weeks, snap frozen in liquid nitrogen and stored at -80°C for later analysis. For each time point, two branches per colony were sampled (4 individuals per replicate aquarium, n = 16), where one branch was used for respirometry assays and selected physiology measurements, and the other for RNA-seq analysis and lipid analysis.

The experimental set-up consisted of 16 (4 aquaria per treatment) flow-through aquaria (80 L) under natural light and a layer of LEE neutral density filters (0.6 ND filter, Panavision, USA), which resulted in daily average photosynthetically active radiation levels of 433.6 ± 8.6 μmole quanta m^-2^ s^-1^ for the light period of the day and a maximum of 859.6 μmole quanta m^-2^ s^-1^ (light levels were measured throughout the duration of the experiment with Odyssey light loggers (Odyssey, New Zealand) that were calibrated against a factory calibrated Li- 192 Li-Cor cosine corrected underwater light sensor (Li-Cor, USA)). Aquaria were supplied with unfiltered seawater which was pumped straight from the reef flat on Heron Island into CO_2_ mixing tanks and then distributed across aquaria under 4 different treatments (see Table 1). Acidification and temperature control of the seawater into the combined pCO_2_ –temperature-controlled system has been described in [24,84]. Briefly, incoming seawater from Heron Island Reef flat was treated in 4 large 10 000 L reservoirs before reaching treatment aquaria. Changes in pCO_2_ were achieved by infusing CO_2_-enriched/or depleted air into the large reservoirs. CO_2_ depletion was attained by passing air through two desiccant columns (Model 106-C, W. A. Hammond Drierite, Australia) filled with soda lime. The pCO_2_ range and dose was controlled by a pCO_2_ analyzer and a custom-built software package (CO_2_ –Pro^TM^, Pro-Oceanus Systems Inc. Canada). Temperature in these large reservoirs was controlled by industrial heater-chillers (HWP017-1BB, Accent Air, Australia). Temperature, pH and light levels were recorded throughout the experiment and total alkalinity (TA) across Pre Industrial (PI), Present Day (PD), Representative Concentration pathways RCP4.5 and RCP8.5 treated aquaria was determined using a Mettler Toledo T50 automated titrator, with 0.1M HCl and 130g seawater samples using the Gran titration method in a two-stage, potentiometric, open-cell titration, following the method of [85]. Acid concentrations and the alkalinity measurements were calibrated at the beginning of each set of water samples being measured using Dickson-certified reference sea water standards (Andrew Dickson, SIO, Oceanic Carbon Dioxide Quality Control). Carbon species concentration (HCO_3_^-^ and CO_3_^2-^) and aragonite state (Ω) were computed for each treatment from measured values of TA, pCO_2_, pH, temperature and salinity and using the CO2SYS program [86]. We used the dissociation constants for carbonate from [87] as refit by [88].

### Measurements of photosynthesis and respirometry rates

Coral branches designated for physiological measurements were used in respirometry assays which generated rates of photosynthesis and respiration for the host symbiont combination (hereby referred to as the ‘holobiont’) following the methods in [89]. Coral branches were dark adapted for at least an hour before respirometry measurements, which were performed after dusk. Branches were placed in 70 cm^3^ clear acrylic chambers with an optode sensor inserted and connected to an Oxy4 v2 system (PreSens, Regensburg, Germany). The chambers were placed within an acrylic container (on top of a magnetic stirrer), which was connected to a water bath keeping the temperature constant throughout the assays at the temperature of 4 different treatments depending on samples being assayed. Chambers were filled with seawater from aquaria undergoing the respective experimental treatments. Photosynthesis vs irradiance curves (P-E curves) analyses were conducted by using a metal halide lamp as the light source, and exposing coral branches in chambers to 0, 10, 20, 55, 110, 925 μmol quanta m^-2^ s^-1^ for 10 min, followed by exposure at 1075 and 1250 μmol quanta m^-2^ s^-1^ for 5 min each, and the run was concluded by a 10 min incubation at 0 μmol quanta m^-2^ s^-1^. Following methods in [23,89] respirometry rates were normalized to symbiont cell number and coral surface area, methods are described below.

Exposure to different light levels enabled the calculation of P-E curve parameters [90] following methods in [89]; dark respiration (R_dark_) estimated from the initial 10 min dark incubation; sub-saturation photosynthetic efficiency (α) derived from the regression line slope of the low irradiance levels (10, 20, 55, 110 μmol quanta m^-2^ s^-1^) in relation to the estimated E_k_, photosynthetic capacity (P_net_ max) was estimated as the greatest rate of oxygen evolution at the high irradiance levels (925, 1075, 1250 μmol quanta m^-2^ s^-1^); and finally, light-enhanced dark respiration (LEDR) was determined from the oxygen consumption in the last dark incubation post irradiance exposure. Following respirometry assays branches were kept in the dark for at least an hour (to allow recovery from respirometry assay that had been conducted after sunset) before they were snap frozen in liquid nitrogen and stored at -80°C for further analysis of *Symbiodinium* population density and pigment and host total water soluble protein.

### Population density and pigment content of *Symbiodinium*

Following the methods of [23] the cell density and pigment content of *Symbiodinium* were measured by removing tissue from coral fragments by air-brushing frozen fragments in 5mL 0.06 M phosphate buffer (pH 6.65). The homogenate was centrifuged at 4000x g for 5 min. The supernatant was removed and the remaining *Symbiodinium* pellet was re-suspended in filtered seawater (0.45 μm) and separated into aliquots that were used for pigment quantification and *Symbiodinium* cell counts. *Symbiodinium* pigment quantification aliquots were centrifuged at 4000x g for 5 min, the supernatant was removed and 1 mL of 100% cold methanol was added to the pellet. The solution was sonicated on ice cold water for 10 min and then centrifuged at 4000x g for 5 min. The supernatant was collected and transferred into a tube. This process was repeated until complete pigment extraction was achieved (when the final supernatant was clear). The total final extracted solution was filtered (0.45 μm) and used for pigment separation in a Shimadzu SCL– 10 HPLC linked to a Shimadzu SPD—M10A photodiode array detector, using the column and method described in [91] with solutions A (methanol: acetonitrile: aquose pyridine, 50:25:25 v:v:v) and B1 (methanol: acetonitrile: acetone, 20:60:20 v:v:v). Standards for methanol extracted pigments chlorophyll *a*, *c*_*2*_. peridinin, β carotene, diatoxanthin and diadinoxanthin were used for quantifying pigments which were normalized per cell. *Symbiodinium* cell counts were estimated using eight randomly selected replicates counted using a haemocytometer (Boeco, Germany) on a Zeiss standard microscope; the counts were normalized to coral surface area in cm^2^, as obtained by twice dipping coral fragments into paraffin wax following the method of [92].

### Measurement of coral growth rates

*A*. *millepora* branch growth/calcification rate was estimated using the buoyant weight method [93]. Coral branch weights for samples across treatments were measured at time zero and after 5 weeks. The branches were suspended by a thin fishing line below a precision balance (Mettler Toledo) and the weight was recorded. The growth/calcification rate was calculated as a relative unit by subtracting the initial weight (g) from the final weight (g) and converting this to a percent change in weight over the course of the experimental period.

### Total water-soluble protein content

To determine total water soluble protein content, the supernatant from the air-brushed tissue was used. The supernatant was analyzed in a SHIMADZU UV 2450 spectrophotometer (Shimadzu, Kyoto, Japan) recording absorbance values at 235 and 280 nm. Total water soluble protein content was determined using the equations of [94].

### Total lipid content

For each of the branches that were assigned for RNA analysis, a small fragment 2 cm from the top of the 8 cm branch was used for analysis of total lipid content. Each fragment was placed into a glass vial, completely immersed in 3ml of chloroform/methanol (2:1) and left overnight at 4°C. The chilled suspension was removed and filtered using a 22μm glass fiber filter and placed in a new 15ml falcon tube. To obtain the total lipid fraction from the chilled suspension methods from [95] were followed. Surface area of the fragment used for lipid content was determined by the paraffin wax method following [92].

### Statistical analysis

All data were tested for normality and homogeneity of variance and where assumptions were violated, the data were corrected by transformations. One way ANOVA was used to determine the effect of corals exposed to different temperature and pCO_2_ scenarios on physiological parameters. Non-parametric equivalent Kruskal-Wallis test was used in cases where assumptions were violated despite transformations. All statistical analyses were performed using STATISTICA 7.0 (Statsoft Inc., Tulsa, USA).

### Total RNA isolation

Total RNA from coral branches was isolated by homogenizing 100 mg coral tissue in 1ml Trizol (Invitrogen) and following the protocol according to the manufacturer’s instructions. The RNA was then extracted once with 1 volume chloroform and then precipitated in ½ volume isopropanol, washed in 1 volume of 75% ethanol and subsequently dissolved in RNAse-free water. These samples were then processed through a 5M LiCl precipitation overnight at -20°C, washed 3 times with 75% ethanol and subsequently dissolved in RNAse-free water. The integrity and quality of total RNA was assessed using a Bioanalyzer (Agilent Technology). Only samples showing intact RNA (RNA Integrity number > 8) were used for RNA-seq analysis.

### RNA-Seq analysis—analyses of differentially expressed gene*s*

Standard RNA-Seq analysis relies on mapping individual short sequence reads to a reference genome or transcriptome and then applying statistical tests to identify differentially expressed genes—DEGs [96–98]. In order to measure changes in the metatranscriptome of the holobiont of *A*. *millepora*, which will include coral, *Symbiodinium* and other potential partner genes, it is challenging to apply the standard RNA-Seq analysis methods. *De novo* assembly of the RNA-Seq data requires large computing resources with high data coverage and is notoriously sensitive to sequencing error and the generation of chimeras in the assembled contigs [99], especially for metatranscriptome data. We, therefore, applied a Differential K-mer Analysis Pipeline (DiffKAP) method that allowed us to identify DEGs between two samples without using a reference [100]. Briefly, the idea of DiffKAP is to break the RNA-Seq datasets into constituent k-mers and normalize this by total k-mer abundance. We then indentify differentially expressed k-mers (DEKs) between the two datasets by comparing the abundance of every unique *k*-mer sequence between datasets. DiffKAP starts by determining an optimal k-mer size by adapting the strategy of [101] to identify the ‘knee point’ in a *k*-mer uniqueness graph. The k-mer size at this point balances the specificity and sensitivity of the information content. DiffKAP applies Jellyfish [102] to perform k-mer counting. The abundance of each k-mer is normalized by dataset size and differentially-expressed k-mers (DEKs) are determined using the following formulas:

$$\text{k}-\text{mer }=\text{ DEK if }\left(\left|O_{T1}–\;O_{T2}\right|\;\geq \;X\right)\text{and }\left(Y\times O_{T1}\leq \;O_{T2}or\;Y\times O_{T2}\leq \;O_{T1}\right)$$

Where *O*_*T1*_ and *O*_*T2*_ represent the normalised k-mer occurrence in datasets 1 and 2 respectively, *X* represents the minimum difference of the k-mer occurrence between the datasets (which is *≥* 3) and *Y* is the minimum fold change of k-mer occurrence between the two datasets (which is *≥* 1.5) required to call a k-mer differentially expressed (DEK). In order to identify a differentially expressed read (DER) a single set of unique reads is obtained by combining all reads in the original datasets and filtering to remove duplicated reads. Then a DER is determined by a strict criterion in order to minimize false positives and this strict criterion is that all constituent k-mers (that is all 85 k-mers) within a read have to be DEK in order for that read to be called a DER. The median k-mer abundance for each DER is calculated for each dataset and the ratio of median k-mer abundance (RoM) is provided as a prediction of gene expression ratio. All DERs are annotated by comparison with the Swiss-Prot database [103], using a strict E-value cut off of ≤ 10^−15^. Annotation of DERs results in a redundant set of differentially expressed genes (DEGs).

We used 1.5-fold change at the DEK stage of the DiffKAP pipline. This cut-off value was chosen to include DEGs of both dinoflagellate and coral origin, as often 2 or lower fold change can be observed in *Symbiodinium* transcript abundance under normal circumstances [104,105]. The DiffKAP program is available from http://appliedbioinformatics.com.au/index.php/DiffKAP.

Equal concentrations of high quality RNA from biological replicates (n = 16) were pooled together and submitted to the Australian Genome Research Fascility Ltd (AGRF) where samples were sequenced using the Illunina TruSeq RNA sample preparation kit and Illumina single end 100bp GA II Sequencing system with 12 samples per lane being sequenced. This resulted in over 6 million reads per replicate (four technical replicates) per sample.

### Validation by Quantitative PCR

Expression patterns of RNA-seq data for13 selected DEGs identified by DiffKAP (S1 Table) were validated with quantitative Polymerase Chain Reaction (qPCR). Primers were designed from RNA-seq data using Primer Experss®Software v3.0 (Applied Biosystems, USA). Total RNA (1000 ng) was reverse transcribed with a Superscript Vilo cDNA synthesis kit (Invitrogen) following manufacturer’s instructions. Transcript levels were determined by qPCR assays using an Eppendorf 5075 (Applied Biosystems, USA) robot to dispense SYBR Green PCR master mix (Applied Biosystems, UK) into 384-well plates, and assays were run in a 7900HT Fast Real-time PCR System (Applied Biosystems, USA). PCR conditions were: initial denaturation of 10 min at 95°C, followed by 45 cycles of 95°C for 15 s and 60°C for 1 min. At the end, a dissociation step was included: 95°C for 2 min, 60°C for 15 s and 95°C for 15 s. The final reaction volume was 10 μl and included 300 nM of primers. All reactions were carried out with two technical replicates. For each candidate gene control (PD) versus high CO_2_ and temperature (RCP8.5) samples (4 replicates and each of the replicates was a pool of 4 biological replicates) were tested, reflecting the conditions under which major physiological changes occurred. A no-template control as well as a no-reverse transcription control was performed for each gene and treatment to ensure that the cDNA samples and PCR reagents did not have DNA contamination. In addition, to ensure the specificity of primers on coral cDNA, primers were tested on cDNA and genomic DNA from *Symbiodinium* sp. as a template in a PCR to ensure no amplification in non-coral DNA. The comparative delta CT method [106] and a maximal PCR efficiency for each gene (E = 2) were used to determine relative quantities of mRNA transcripts from each sample. Each value was normalized to two reference genes, adenosyl-homocysteinase (AdoHcyase) and ribosomal protein L7 (Rpl7). The selection of reference genes for this experiment was done by using a pool of reference genes (S1 Table) and analyzing their expression stability using the GeNorm software [107]. For this study the most stable expression was found for AdoHcyase and Rpl7 (M value = 0.235) and a minimum of two reference genes was recommended (V2/3 < 0.15). The real-time dissociation curve was used to check for the presence of a unique PCR product. Relative expression values for each gene were calculated by showing a ratio of Treatment relative expression over Control relative expression. The results of qPCR and k-mer (DiffKAP method) analyses are presented on a log_2_ scale. To test for significance of the difference in expression levels of each gene, from quantitative real-time PCR comparing present day and RCP8.5 conditions, a Welch t-test was used.

### Taxonomic composition of metatranscriptomes

In this study we used publicly available datasets, including expressed sequence tags (ESTs), genome and transcriptome sequences. To determine the taxonomic composition of each metatranscriptome, short reads from each of the analyzed samples were aligned to sequences from Genbank databases (accessed May 2015; that included bacterial, environmental, invertebrate, plant, and viral nucleotide sequences; *A*. *digitifera* genome; human genome; and *Symbiodinium* ESTs); then additional *Symbiodinium* ESTs (Joint Genome Institute, University of California); the *A*. *millepora* transcriptome [28,108]; the *A*. *hyacinthus*, *A*. *tenuis* and *Porites astreoides* transcriptomes (Eli Meyer, Mikhail Matz, *et al*., unpublished data, www.bio.utexas.edu/research/matz_lab/); *Symbiodinium minutum* nuclear genome [109] and *de novo Symbiodinium* transcriptomes [61]. Database alignments were carried out using the Nucleotide-Nucleotide BLAST software application (BLASTn, version 2.2.27+), specifying a word size of 14 and E-values ≤ 10^−15^.

### Gene ontology enrichment analyses

We applied gene ontology (GO) enrichment analyses to the list of DEGs for each treatment/control comparison. GO enrichment analyses and pathway analyses were performed using the database for annotation, visualization and integrated discovery (DAVID) and software tools therein, to identify enriched biological themes and **KEGG** (Kyoto Encyclopedia of Genes and Genomes) pathways [110,111]. DAVID uses the Fisher’s Exact Test to ascertain statistically significant gene enrichment for a particular pathway, and significant processes were selected based on a corrected P-value smaller than 0.05. In order to generate networks of highly interconnected proteins for the DEG list of the RCP8.5/ PD comparison, we used the STRING (Search Tool for the Retrieval of Interacting Genes, Heidelberg, Germany) 9.1 database [112]. We examined clustering of protein interactions with high confidence scores (≥ 0.7).

## Supporting Information

S1 Fig Number of differentially expressed genes (DEGs). Venn diagrams showing numbers and overlap in DEGs in *Acropora millepora* holobiont exposed to Pre Industrial (PI) vs Present Day (PD) conditions, Representative Concentration Pathway RCP4.5 vs PD conditions and RCP8.5 vs PD conditions. Venn diagram represents the overlaps between DEGs among treatments.
(EPS)

S2 Fig Taxonomic composition. Taxonomic composition of *Acropora millepora* holobiont samples exposed to (**A**) Pre Industrial (PI) (**B**) Present Day (PD) (**C**) Representative Concentration Pathway RCP4.5 and (**D**) RCP8.5 conditions. Taxonomic composition was obtained by aligning short read data from RNA-seq to publicly available sequence databases.
(EPS)

S3 Fig Correlation of gene expression data between RNA-seq and quantitative PCR (qPCR). Log2 fold change in relative gene expression between values obtained from RNA-seq DiffKAP approach with expression values obtained using qPCR. 13 genes were arbitrarily chosen based on either high up or down regulation in treatment versus present day conditions.
(EPS)

S1 File Gene enrichment analysis. Gene enrichment analysis (P<0.05) of biological processes (BP) and molecular functions (MF) enriched under all 3 treatments (Pre Industrial, Representative Concentration pathway RCP4.5 and RCP8.5) as compared to Present Day conditions.
(XLSX)

S2 File Gene enrichment analysis of genes enriched under all 3 treatments. Gene enrichment analysis (P<0.05) of biological processes and molecular functions enriched under all 3 treatments (Pre Industrial, Representative Concentration pathway RCP4.5 and RCP8.5) as compared to Present Day conditions. Enrichment analysis was performed on 210 genes found in core from S1 Fig.
(XLSX)

S1 Table Up and down regulated genes. The UniProt IDs and descriptions of 30 top up regulated and 30 top down regulated genes involved in cellular processes for *A*. *millepora* holobiont exposed to temperature and pCO2 levels predicted by the IPCC for Pre Industrial, Representative Concentration pathway RCP4.5 and RCP8.5 conditions as compared to Present Day conditions.
(PDF)

S2 Table List of candidate genes used in qPCR expression analysis. 13 genes were arbitrarily chosen based on either high up or down regulation in treatment versus present day conditions and a pool of 4 candidate reference genes.
(PDF)

S3 Table Gene expression under Representative Concentration Pathway (RPC) 8.5 scenario. The UniProt IDs and descriptions of genes involved in cellular processes for *A*. *millepora* holobiont exposed to increased temperature and pCO2 levels predicted by the RPC 8.5 scenario.
(PDF)

## References

[1] Hoegh-Guldberg O, Bruno JF (2010) The Impact of Climate Change on the World’s Marine Ecosystems. Science 328: 1523–1528. 10.1126/science.1189930 20558709

[2] Hoegh-Guldberg O, Cai R, Poloczanska ES, Brewer PG, Sundby S, Hilmi V et al (2014) The Ocean In: Barros VR, Field CB, Dokken DJ, Mastrandrea MD, Mach KJ et al, editors. Climate Change 2014: Impacts, Adaptation and Vulnerability Part B: Regional Aspects Contribution of Working Group II to the Fifth Assessment Report of the Intergovernmental Panel on Climate Change. Cambridge, United Kingdom and New York, NY, USA: Cambridge University Press.

[3] Hoegh-Guldberg O, Mumby P, Hooten AJ, Steneck RS, Greenfield P, Gomez E et al (2007) Coral reefs under rapid climate change and ocean acidification. Science 318: 1737–1742. 18079392 10.1126/science.1152509

[4] Pandolfi JM, Connolly SR, Marshall DJ, Cohen AL (2011) Projecting coral reef futures under global warming and ocean acidification. Science 333: 418–422. 10.1126/science.1204794 21778392

[5] Moberg F, Folke C (1999) Ecological goods and services of coral reef ecosystems. Ecological Economics 29: 215–233.

[6] Lough JM (2008) 10^th^ Anniversary review: a changing climate for coral reefs. Journal of Environmental Monitoring 10: 21–61. 10.1039/b714627m 18175015

[7] Baker AC, Glynn PW, Reigl B (2008) Climate change and coral bleaching: An ecological assessment of long-term impacts, recovery trends and future outlook. Estuarine Coastal and Shelf Science 80: 435–471.

[8] Hughes TP, Baird AH, Bellwood DR, Card M, Connolly SR, Folke C et al (2003) Climate change, human impacts and the resilience of coral reefs. Science 301: 929–933. 12920289 10.1126/science.1085046

[9] Walther GR, Post E, Convey P, Menzel A, Parmesan C, Beebee TJC et al (2002) Ecological responses to recent climate change. Nature 416: 389–395. 11919621 10.1038/416389a

[10] Baird AH, Marshall PA (2002) Mortality, growth and reproduction in scleractinian corals following bleaching on the Great Barrier Reef. Marine Ecology Progress Series 237: 133–141.

[11] Mendes J, Woodley J (2002) Effect of the 1995–1996 bleaching event on polyp tissue depth, growth, reproduction and skeletal band fromation in *Montastrea annularis* . Marine Ecology Progress Series 235: 93–102.

[12] Loya Y, Sakai K, Yamazato K, Nakano Y, Sambali H, van Woesik R (2001) Coral bleaching: the winners and losers. Ecology Letters 4: 122–131.

[13] Pelejero C, Calvo E, Hoegh-Guldberg O (2010) Paleo-perspective on ocean acidification. Trends in Ecology & Evolution 25: 332–345. 20356649 10.1016/j.tree.2010.02.002

[14] Fabricius KE, Langdon C, Uthicke S, Humphrey C, Noonan S, De’ath G et al (2011) Losers and winners in coral reefs acclimatized to elevated carbon dioxide concentrations. Nature Clim Change 1: 165–169.

[15] Inoue S, Kayanne H, Yamamoto S, Kurihara H (2013) Spatial community shift from hard to soft corals in acidified water. Nature Clim Change 3: 683–687.

[16] Doney SC, Fabry VJ, Feely RA, Kleypas JA (2009) Ocean acidification:the other CO_2_ problem. Annual Review of Marine Science 1: 169–192. 21141034 10.1146/annurev.marine.010908.163834

[17] Kleypas JA, McManus JW, Menez LAB (1999) Environmental limits to coral reef development: where do we draw the line? American Zoologist 39: 146–159.

[18] Cohen AL, McCorkle DC, de Putron S, Glenn GA, Rose KA (2009) Morphological and compositional changes in the skeletons of new coral recruits reared in acidified seawater: insights into the biomineralization response to ocean acidification. Geochemistry Geophysics Geosystems 10: Q07005.

[19] Kroeker KJ, Kordas RL, Crim R, Hendriks IE, Ramajo L, Singh GS, et al (2013) Impacts of ocean acidification on marine organisms: quantifying sensitivities and interaction with warming. Global Change Biology 19: 1884–1896. 10.1111/gcb.12179 23505245 PMC3664023

[20] Anthony KRN, Kline DI, Diaz-Pulido G, Dove S, Hoegh-Guldberg O (2008) Ocean acidification causes bleaching and productivity loss in coral reef builders. Proceedings of the National Academy of Sciences of the United States of America 105: 17442–17446. 10.1073/pnas.0804478105 18988740 PMC2580748

[21] Munday P, Dixon D, Donelson J, Jones G, Pratchett M, Devitsina G, et al (2009) Ocean acidification impairs olfactory discrimination and homing ability of a marine fish. Proceeding of the National Academy of Science USA 106: 1848. 10.1073/pnas.0809996106 PMC2644126 19188596

[22] Pörtner HO (2008) Ecosystem effects of ocean acidification in times of ocean warming: a physiologist’s view. Marine Ecology Progress Series 373: 203–217.

[23] Kaniewska P, Campbell PR, Kline DI, Rodriguez-Lanetty M, Miller DJ, et al (2012) Major Cellular and Physiological Impacts of Ocean Acidification on a Reef Building Coral. Plos One 7: e34659 10.1371/journal.pone.0034659 22509341 PMC3324498

[24] Dove SG, Kline DI, Pantos O, Angly FE, Tyson GW, Hoegh-Guldberg O (2013) Future reef decalcification under a business-as-usual CO2 emission scenario. Proceedings of the National Academy of Sciences 110: 15342–15347. 10.1073/pnas.1302701110 PMC3780867 24003127

[25] Edmunds PJ, Brown D, Moriarty V (2012) Interactive effects of ocean acidification and temperature on two scleractinian corals from Moorea, French Polynesia. Global Change Biology 18: 2173–2183.

[26] Rodolfo-Metalpa R, Houlbrèque F, Tambutte E, Boisson F, Baggini C, Patti FP, et al (2011) Coral and mollusc resistance to ocean acidification adversely affected by warming. Nature Climate Change 1: 308–312.

[27] Yara Y, Vogt M, Fujii M, Yamano H, Hauri C, Steinacher M, et al (2012) Ocean acidification limits temperature-induced poleward expansion of coral habitats around Japan. Biogeosciences 9: 4955–4968.

[28] Moya A, Huisman L, Ball EE, Hayward DC, Grasso LC, Chua CM, et al (2012) Whole Transcriptome Analysis of the Coral Acropora millepora Reveals Complex Responses to CO2-driven Acidification during the Initiation of Calcification. Molecular Ecology 21: 2440–2454. 10.1111/j.1365-294X.2012.05554.x 22490231

[29] Vidal-Dupiol J, Zoccola D, Tambutté E, Grunau C, Cosseau C, Smith KM et al (2013) Genes Related to Ion-Transport and Energy Production Are Upregulated in Response to CO2-Driven pH Decrease in Corals: New Insights from Transcriptome Analysis. Plos One 8: e58652 10.1371/journal.pone.0058652 23544045 PMC3609761

[30] Barshis DJ, Ladner JT, Oliver TA, Seneca FO, Traylor-Knowles N, et al (2013) Genomic basis for coral resilience to climate change. Proceedings of the National Academy of Sciences 110: 1387–1392. 10.1073/pnas.1210224110 PMC3557039 23297204

[31] DeSalvo MK, Sunagawa S, Voolstra CR, Medina M (2010) Transcriptomic responses to heat stress and bleaching in the elkhorn coral *Acropora palmata* . Marine Ecology Progress Series 402: 97–113.

[32] DeSalvo MK, Voolstra CR, Sunagawa S, Schwarz JA, Stillman JH, Palumbi SR (2008) Differential gene expression during thermal stress and bleaching in the Caribbean coral Montastraea faveolata. Mol Ecol 17: 3952–3971. 10.1111/j.1365-294X.2008.03879.x 18662230

[33] Maor-Landaw K, Karako-Lampert S, Ben-Asher HW, Goffredo S, Falini G, Dubinsky Z, et al (2014) Gene expression profiles during short-term heat stress in the red sea coral Stylophora pistillata. Global Change Biology 20: 3026–3035. 10.1111/gcb.12592 24706387

[34] Rodriguez-Lanetty M, Harii S, Hoegh-Guldberg OVE (2009) Early molecular responses of coral larvae to hyperthermal stress. Molecular Ecology 18: 5101–5114. 10.1111/j.1365-294X.2009.04419.x 19900172

[35] Rodolfo-Metalpa R, Martin S, Ferrier-Pages C, Gatusso J (2010) Response of the temperate coral *Cladocora caespitosa* to mid- and long-term exposure to pCO_2_ and temperature levels projected for the year 2100 AD. Biogeosciences 7: 289–300.

[36] Weis VM (2008) Cellular mechanisms of Cnidarian bleaching: stress causes the collapse of symbiosis. Journal of Experimental Biology 211: 3059–3066. 10.1242/jeb.009597 18805804

[37] Chan NCS, Connolly SR (2012) Sensitivity of coral calcification to ocean acidification: a meta-analysis. Global Change Biology 19: 282–290. 10.1111/gcb.12011 23504739

[38] Harvey B, Gwynn-Jones D, Moore P (2013) Meta-analysis reveals complex marine biological responses to the interactive effects of ocean acidification and warming. Ecology and Evolution 3: 1016–1030. 10.1002/ece3.516 23610641 PMC3631411

[39] Kroeker K, Kordas R, Crim R, Singh G (2010) Meta-analysis reveals negative yet variable effects of ocean acidification on marine organisms. Ecology Letters 13: 1419–1434. 10.1111/j.1461-0248.2010.01518.x 20958904

[40] Obara M, Szeliga M, Albrecht J (2008) Regulation of pH in the mammalian central nervous system under normal and pathological conditions: Facts and hypotheses. Neurochemistry International 52: 905–919. 18061308 10.1016/j.neuint.2007.10.015

[41] McCulloch M, Falter J, Trotter J, Montagna P (2012) Coral resilience to ocean acidification and global warming through pH up-regulation. Nature Clim Change 2: 623–627.

[42] Venn AA, Tambutté E, Lotto S, Zoccola D, Allemand D, Tambutté S(2009) Imaging intracellular pH in a reef coral and symbiotic anemone. Proceedings of the National Academy of Sciences 106: 16574–16579. 10.1073/pnas.0902894106 PMC2757848 19720994

[43] Venn AA, Tambutté E, Holcomb M, Laurent J, Allemand D, Tambutté S (2013) Impact of seawater acidification on pH at the tissue–skeleton interface and calcification in reef corals. Proceedings of the National Academy of Sciences 110: 1634–1639. 10.1073/pnas.1216153110 PMC3562847 23277567

[44] Cohen AL, Holcomb M (2009) Why Corals Care about Ocean Acidification: Uncovering the Mechanism. Oceanography 22: 118–127.

[45] Cooper TF, Lai M, Ulstrup KE, Saunders SM, Flematti GR, Radford B, et al (2011) *Symbiodinium* Genotypic and Environmental Controls on Lipids in Reef Building Corals. Plos One 6: e20434 10.1371/journal.pone.0020434 21637826 PMC3102723

[46] Fine M, Tchernov D (2007) Scleractinian Coral Species Survive and Recover from Decalcification. Science 315: 1811 17395821 10.1126/science.1137094

[47] Schoepf V, Grottoli AG, Warner ME, Cai W-J, Melman TF, Hoadley KD, et al (2013) Coral Energy Reserves and Calcification in a High-CO2 World at Two Temperatures. Plos One 8: e75049 10.1371/journal.pone.0075049 24146747 PMC3795744

[48] Ward S (1995) Two patterns of energy allocation for growth, reproduction and lipid storage in the scleractinian coral Pocillopora damicornis. Coral Reefs 14: 87–90.

[49] Gibbin E, Putnam H, Davy S, Gates RD (2014) Intracellular pH and its response to CO_2_ driven seawater acidification in symbiotic versus non-symbiotic cells. Journal of Experimental Biology 217: 1963–1969. 10.1242/jeb.099549 24625648

[50] Falkowski PG, Dubinsky Z (1981) Light-shade adaptation of Stylophora pistillata, a hermatypic coral from the Gulf of Eliat. Nature 289: 172–174.

[51] Baroli I, Do AD, Yamane T, Niyogi KK (2003) Zexanthin accumulation in the absence of a functional xanthophyll cycle protects *Chlamydomonas reinhardtii* from photooxidative stress. The Plant Cell 15: 992–1008. 12671093 10.1105/tpc.010405 PMC152344

[52] Chaumont D, Thepenier C (1995) Carotenoid content in growing cells of *Haematococcus pluvalis* during a sunlight cycle. Journal of Applied Phycology 7: 529–537.

[53] Baroli I, Gutman BL, Ledford HK, Shin JW, Chin BL, Havaux M, et al (2004) Photo-oxidative stress in the xanthophyll-deficient mutant of *Chlamydomonas* . Journal of Biological Chemistry 279: 6337–6344. 14665619 10.1074/jbc.M312919200

[54] Brown BE, Ambarsari I, Warner ME, Fitt WK, Dunne RP, Gibb SW, et al (1999) Diurnal changes in photochemical efficiency and xantophyll concentrations in shallow water reef corals: evidence for photoinhibition and photoprotection. Coral Reefs 18: 99–105.

[55] Muller P, Li XP, Niyogi KK (2001) Non-Photochemical Quenching. A Response to Excess Light Energy. Plant Physiology 125: 1558–1566. 11299337 10.1104/pp.125.4.1558 PMC1539381

[56] Ulstrup KE, Hill R, van Oppen MJH, Larkum AWD, Ralph PJ (2008) Seasonal variation in the photo-physiology of homogeneous and heterogeneous Symbiodinium consortia in two scleractinian corals. Marine Ecology-Progress Series 361: 139–150.

[57] Demmig-Adams B, Adams W (1996) The role of xanthophyll cycle carotenoids in the protection of photosynthesis. Trends in Plant Science 1: 21–26.

[58] Rosenberg E, Koren O, Reshef L, Efrony R, Zilber-Rosenberg I (2007) The role of microorganisms in coral health, disease and evolution. Nature Reviews Microbiology 5: 355–362. 17384666 10.1038/nrmicro1635

[59] Yarden O, Ainsworth TD, Roff G, Leggat W, Fine M, Hoegh Guldberg O(2007) Increased prevalence of ubiquitous ascomycetes in an acropoid coral (Acropora formosa) exhibiting symptoms of brown band syndrome and skeletal eroding band disease. Applied and Environmental Microbiology 73: 2755–2757. 17308192 10.1128/AEM.02738-06 PMC1855611

[60] Rohwer F, Seguritan V, Azam F, Knowlton N (2002) Diversity and distribution of coral-associated bacteria. Marine Ecology Progress Series 243: 1–10.

[61] Rosic N, Ling EYS, Chan CKK, Lee HC, Kaniewska P, Edwards D, et al (2015) Unfolding the secrets of coral-algal symbiosis. ISME J 9: 844–856. 10.1038/ismej.2014.182 25343511 PMC4817714

[62] Martin DE, Soulard A, Hall MN (2004) TOR Regulates Ribosomal Protein Gene Expression via PKA and the Forkhead Transcription Factor FHL1. Cell 119: 969–979. 15620355 10.1016/j.cell.2004.11.047

[63] Nozawa Y (2012) Annual Variation in the Timing of Coral Spawning in a High-Latitude Environment: Influence of Temperature. The Biological Bulletin 222: 192–202. 22815368 10.1086/BBLv222n3p192

[64] Guppy M, Withers P (1999) Metabolic depression in animals: physiological perspectives and biochemical generalizations. Biological Reviews 74. 10.1017/s0006323198005258 10396183

[65] Eckel-Mahan K, Sassone-Corsi P (2013) Metabolism and the Circadian Clock Converge. Physiological Reviews 93: 107–135. 10.1152/physrev.00016.2012 23303907 PMC3781773

[66] Adamovich Y, Rousso-Noori L, Zwighaft Z, Neufeld-Cohen A, Golik M, Kraut-Cohen J, et al (2010) Circadian Clocks and Feeding Time Regulate the Oscillations and Levels of Hepatic Triglycerides. Cell Metabolism 19: 319–330. 10.1016/j.cmet.2013.12.016 PMC4261230 24506873

[67] Levy O, Kaniewska P, Alon S, Eisenberg E, Karako-Lampert S, Bay LK, et al (2011) Complex diel cycles of gene expression in coral-algal symbiosis. Science 331: 175 10.1126/science.1196419 21233378

[68] Mazzoccoli G, Pazienza V, Vinciguerra M (2012) Clock genes and clock-controlled genes in the regulation of metabolic rhythms. Chronobiology International 29: 227–251. 10.3109/07420528.2012.658127 22390237

[69] Sha L, MacIntyre L, Machell JA, Kelly MP, Porteous DJ, Brandon NJ, et al (2012) Transcriptional regulation of neurodevelopmental and metabolic pathways by NPAS3. Mol Psychiatry 17: 267–279. 10.1038/mp.2011.73 21709683

[70] Kültz D (2005) Molecular and evolutionary basis of the cellular stress response. Annual Review of Physiology 67: 225–257. 15709958 10.1146/annurev.physiol.67.040403.103635

[71] Perkins ND (2007) Integrating cell-signalling pathways with NF-[kappa]B and IKK function. Nat Rev Mol Cell Biol 8: 49–62. 17183360 10.1038/nrm2083

[72] Bourne DG, Iida Y, Uthicke S, Smith-Keune C (2008) Changes in coral-assiciated microbial communities during a bleaching event. ISME J 2: 350–363. 18059490 10.1038/ismej.2007.112

[73] Vega Thurber R, Willner-Hall D, Rodriguez-Mueller B, Desnues C, Edwards R, Angly F, et al (2009) Metagenomic analysis of stressed coral holobionts. Environmental Microbiology 11: 2148–2163. 10.1111/j.1462-2920.2009.01935.x 19397678

[74] Ciechanover A (2005) Proteolysis: from the lysosome to ubiquitin and the proteasome. Nat Rev Mol Cell Biol 6: 79–87. 15688069 10.1038/nrm1552

[75] Downs C, Ostrander G, Rougee L, Rongo T, Knutson S, Williams DE, et al (2012) The use of cellular diagnostics for identifying sub-lethal stress in reef corals. Ecotoxicology 21: 768–782. 10.1007/s10646-011-0837-4 22215560

[76] DeSalvo MK, Estrada A, Sunagawa S, Medina M (2012) Transcriptomic responses to darkness stress point to common coral bleaching mechanisms. Coral Reefs 31: 215–228.

[77] Cui Y, Niziolek PJ, MacDonald BT, Zylstra CR, Alenina N, Robinson DR, et al (2011) Lrp5 functions in bone to regulate bone mass. Nat Med 17: 684–691. 10.1038/nm.2388 21602802 PMC3113461

[78] Bergh J, Shao Y, Puente E, Duncan R, Farach-Carson M (2006) Osteoblast Ca^2+^ permeability and voltage-sensitive Ca^2+^ channel expression is temporally regulated by 1,25-dihydroxyvitamin D_3_ . American Journal of Physiology—Cell Physiology 290: C822–C831. 16221734 10.1152/ajpcell.00403.2005

[79] Shao Y, Alicknavitch M, Farach-Carson MC (2005) Expression of voltage sensitive calcium channel (VSCC) L-type Cav1.2 (α1C) and T-type Cav3.2 (α1H) subunits during mouse bone development. Developmental Dynamics 234: 54–62. 16059921 10.1002/dvdy.20517

[80] Allemand D, Tambutte E, Zoccola D, Tambutte S (2011) Coral calcification, cells to reefs In: Dubinsky Z, Stambler N, editors. Coral reefs: an ecosystem in transition. London, New York: Springer pp. 119–150.

[81] Furla P, Allemand D, Orsenigo M (2000) Involvement of H^+^ -ATPase and carbonic anhydrase in inorganic carbon uptake for endosymbiont photosynthesis. American Journal of Physiology: Regulatory Integrative Comparative Physiology 278: R870–R881. 10.1152/ajpregu.2000.278.4.R870 10749774

[82] Supuran C (2008) Carbonic anhydrases—an overview. Current Pharmaceutical Design 14: 603–614. 18336305 10.2174/138161208783877884

[83] Jackson SP, Bartek J (2009) The DNA-damage response in human biology and disease. Nature 461: 1071–1078. 10.1038/nature08467 19847258 PMC2906700

[84] Reyes-Nivia C, Diaz-Pulido G, Kline D, Guldberg O-H, Dove S (2013) Ocean acidification and warming scenarios increase microbioerosion of coral skeletons. Global Change Biology 19: 1919–1929. 10.1111/gcb.12158 23505093

[85] Dickson AG, Afghan JD, Anderson GC (2003) Reference materials for oceanic CO_2_ analysis: a method for the certification of total alkalinity. Marine Chemistry 80: 185–197.

[86] Lewis E, Wallace DWR (1998) Program developed for CO2 system calculations. In: Carbon Dioxide Information Analysis Center ORNL, US., editor. Oak Ridage, TN: Department of Energy.

[87] Mehrbach C, Culberson CH, Hawley JE, Pytkowicz RM (1973) Measurement of the apparent dissociation constants of carbonic acid in seawater at atmospheric pressure. Limnology and Oceanography 18: 897–907.

[88] Dickson AG, Millero FJ (1987) A comparison of the equilibrium constants for the dissociation of carbonic acid in seawater media. Deep Sea Research 34: 1733–1743.

[89] Crawley A, Kline DI, Dunn S, Anthony KRN, Dove S (2010) The effect of ocean acidification on symbiont photorespiration and productivity in *Acropora formosa* . Global Change Biology 15: 851–197.

[90] Barnes D, Chalker B (1990) Calcification and photosynthesis in reef-building corals and algae In: D Z., editor. Ecosystems of the World: Vol 25, Coral Reefs. Amsterdam: Elsevier Science pp. 109–131.

[91] Zapata M, Rodriguez F, Garrrido JL (2000) Separation of chlorophylls and carotenoids from marine phytoplankton: a new HPLC method using a reversed-phase C8 column and pyridine-containing mobile phases. Marine Ecology Progress Series 195: 29–45.

[92] Stimson J, Kinzie RA (1991) The temporal pattern and rate of release of zooxanthellae from the reef coral *Pocillopora damicornis* (Linnaeus) under nitrogen-enrichment and control conditions. Journal of Experimental Marine Biology and Ecology 153: 63–74.

[93] Spencer-Davies P (1989) Short-term growth measurements of coral growth using an accurate buoyant weighing technique. Marine Biology 101: 389–395.

[94] Whitaker JR, Granum PE (1980) An absolute method for protein determination based on differences in absorbance at 235 and 280 nm. Analytical Biochemistry 109: 156–159. 7469012 10.1016/0003-2697(80)90024-x

[95] Dunn SR, Thomas MC, Nette GW, Dove SG (2012) A Lipidomic Approach to Understanding Free Fatty Acid Lipogenesis Derived from Dissolved Inorganic Carbon within Cnidarian-Dinoflagellate Symbiosis. Plos One 7: e46801 10.1371/journal.pone.0046801 23115631 PMC3480374

[96] Robinson MD, Oshlack A (2010) A scaling normalization method for differential expression analysis of RNA-seq data. Genome Biology 11: R25 10.1186/gb-2010-11-3-r25 20196867 PMC2864565

[97] Trapnell C, Roberts A, Goff L, Pertea G, Kim D, Kelley DR, et al (2012) Differential gene and transcript expression analysis of RNA-seq experiments with TopHat and Cufflinks. Nat Protocols 7: 562–578. 10.1038/nprot.2012.016 22383036 PMC3334321

[98] Wang L, Feng Z, Wang X, Wang X, Zhang X (2010) DEGseq: an R package for identifying differentially expressed genes from RNA-seq data. Bioinformatics 26: 136–138. 10.1093/bioinformatics/btp612 19855105

[99] Martin JA, Wang Z (2011) Next-generation transcriptome assembly. Nat Rev Genet 12: 671–682. 10.1038/nrg3068 21897427

[100] Rosic N, Kaniewska P, Chan CKK, Ling EYS, Edwards D, Dove S, et al (2014) Early transcriptional changes in the reef-building coral *Acropora aspera* in response to thermal and nutrient stress. BMC Genomics 15: 1052 10.1186/1471-2164-15-1052 25467196 PMC4301396

[101] Kurtz S, Narechania A, Stein J, Ware D (2008) A new method to compute K-mer frequencies and its application to annotate large repetitive plant genomes. BMC Genomics 9: 517 10.1186/1471-2164-9-517 18976482 PMC2613927

[102] Marçais G, Kingsford C (2011) A fast, lock-free approach for efficient parallel counting of occurrences of k-mers. Bioinformatics 27: 764–770. 10.1093/bioinformatics/btr011 21217122 PMC3051319

[103] Boutet E, Lieberherr D, Tognolli M, Schneider M, Bairoch A (2007) UniProtKB/Swiss-Prot. Methods in molecular biology 406: 89–112. 18287689 10.1007/978-1-59745-535-0_4

[104] Leggat W, Seneca F, Wasmund K, Ukani L, Yellowlees D, Ainsworth TD (2011) Differential Responses of the Coral Host and Their Algal Symbiont to Thermal Stress. Plos One 6: e26687 10.1371/journal.pone.0026687 22039532 PMC3200360

[105] Rosic N, Pernice M, Dove S, Dunn S, Hoegh-Guldberg O (2011) Gene expression profiles of cytosolic heat shock proteins Hsp70 and Hsp90 from symbiotic dinoflagellates in response to thermal stress: possible implications for coral bleaching. Cell Stress and Chaperones 16: 69–80. 10.1007/s12192-010-0222-x 20821176 PMC3024090

[106] Walker NJ (2002) A Technique Whose Time Has Come. Science 296: 557–559. 11964485 10.1126/science.296.5567.557

[107] Vandesompele J, De Preter K, Pattyn F, Poppe B, Van Roy N, De Paepe A, et al (2002) Accurate normalization of real-time quantitative RT-PCR data by geometric averaging of multiple control genes. Genome Biology 3: 6906–6914. 10.1186/gb-2002-3-7-research0034 PMC126239 12184808

[108] Meyer E, Aglyamova G, Wang S, Buchanan-Carter J, Abrego D, Colbourne J, et al (2009) Sequencing and de novo analysis of a coral larval transcriptome using 454 GSFlx. BMC Genomics 10: 219 10.1186/1471-2164-10-219 19435504 PMC2689275

[109] Shoguchi E, Shinzato C, Kawashima T, Gyoja F, Mungpakdee S, Koyangi R, et al (2013) Draft assembly of the Symbiodinium minutum nuclear genome reveals dinoflagellate gene structure. Current Biology 23: 1399–1408. 10.1016/j.cub.2013.05.062 23850284

[110] Huang DW, Sherman BT, Lempicki RA (2008) Systematic and integrative analysis of large gene lists using DAVID bioinformatics resources. Nat Protocols 4: 44–57. 10.1038/nprot.2008.211 19131956

[111] Huang DW, Sherman BT, Lempicki RA (2009) Bioinformatics enrichment tools: paths toward the comprehensive functional analysis of large gene lists. Nucleic Acids Research 37: 1–13. 10.1093/nar/gkn923 19033363 PMC2615629

[112] Szklarczyk D, Franceschini A, Kuhn M, Simonovic M, Roth A, Minguez P, et al (2011) The STRING database in 2011: functional interaction networks of proteins, globally integrated and scored. Nucleic Acids Research 39: D561–D568. 10.1093/nar/gkq973 21045058 PMC3013807

