# Supplementary material for: Transcriptomic Changes in Coral Holobionts Provide Insights into Physiological Challenges of Future Climate and Ocean Change
Source: PLoS One. 2015 Oct 28;10(10):e0139223. doi: 10.1371/journal.pone.0139223 (PMC4624983; doi:10.1371/journal.pone.0139223)
Supplement: S1 Table — The UniProt IDs and descriptions of 30 top up regulated and 30 top down regulated genes involved in cellular processes for A. millepora holobiont exposed to temperature and pCO2 levels predicted by the IPCC for Pre Industrial, Representative Concentration pathway RCP4.5 and RCP8.5 conditions as compared to Present Day conditions. (PDF) [file pone.0139223.s006.pdf]

S1 Table The UniProt IDs and descriptions of 30 top up regulated and 30 top down regulated genes involved in cellular processes for *A. millepora* holobiont exposed to temperature and pCO2 levels predicted by the IPCC for Pre Industrial, Representative Concentration pathway RCP4.5 and RCP8.5 conditions as compared to Present Day conditions.

UniProt ID	Hit description	Taxonomic origin	Expression direction
Pre Industrial			
Q9MSC2	Photosystem Q(B) protein	*Symbiodinium*	up
Q95P04	GFP-like non-fluorescent chromoprotein	coral	up
P08537	Tubulin alpha chain	coral	up
C5NSL2	Bandaporin	coral	up
Q2JRJ5	Photosystem II D2 protein	*Symbiodinium*	up
O09005	Sphingolipid delta(4)-desaturase DES1	*Symbiodinium*	up
Q91XQ0	Dynein heavy chain 8, axonemal	coral	up
C9EIC7	Urticinatoxin	coral	up
Q9VQX4	Nicotinate phosphoribosyltransferase	coral	up
P35571	Glycerol-3-phosphate dehydrogenase, mitochondrial	coral	up
Q94572	Tubulin alpha-3 chain	coral	up
P28826	Meprin A subunit beta	coral	up
P51753	Photosystem II 44 kDa reaction center protein	*Symbiodinium*	up
Q95VF7	Profilin-1B	coral	up
Q8VHE6	Dynein heavy chain 5, axonemal	coral	up
O70260	E3 SUMO-protein ligase PIAS3	coral	up
Q9VSA3	Probable medium-chain specific acyl-CoA dehydrogenase, mitochondrial	coral	up
Q9MUV3	Cytochrome b6	*Symbiodinium*	up
Q86FQ0	Cytolysin Src-1	coral	up
P82252	B(0,+)-type amino acid transporter 1	coral	up
Q9QXK7	Cleavage and polyadenylation specificity factor subunit 3	coral	up
P04961	Proliferating cell nuclear antigen	coral	up
Q9JHX2	Transcription factor Sp5	coral	up
Q8CFI7	DNA-directed RNA polymerase II subunit RPB2	coral	up
Q91Z80	Liprin-alpha-4	coral	up
Q6PH19	Glycine amidinotransferase, mitochondrial	coral	up
Q62674	Sodium/nucleoside cotransporter 1	coral	up

O78511	Photosystem II CP47 chlorophyll apoprotein	*Symbiodinium*	up
P51650	Succinate-semialdehyde dehydrogenase, mitochondrial	coral	up
Q0HA38	Tetratricopeptide repeat protein 21B	coral	up
P51874	Peridinin-chlorophyll a-binding protein, chloroplastic	*Symbiodinium*	down
Q59754	Pyruvate, phosphate dikinase	*Symbiodinium*	down
Q41406	Ribulose bisphosphate carboxylase	*Symbiodinium*	down
P22983	Pyruvate, phosphate dikinase	*Symbiodinium*	down
P51644	ADP-ribosylation factor 4	*Symbiodinium*	down
P40945	ADP-ribosylation factor 2	*Symbiodinium*	down
Q64MT2	Adenosylhomocysteinase	coral	down
P10878	Tubulin beta chain	*Symbiodinium*	down
P11857	Tubulin beta chain	*Symbiodinium*	down
O61065	Ribonucleoside-diphosphate reductase large chain	*Symbiodinium*	down
Q01604	Phosphoglycerate kinase	*Symbiodinium*	down
Q9FM01	Probable UDP-glucose 6-dehydrogenase 2	*Symbiodinium*	down
P11941	Lysozyme C II	coral	down
Q9SYM5	Probable rhamnose biosynthetic enzyme 1	*Symbiodinium*	down
Q25761	ADP-ribosylation factor 1	*Symbiodinium*	down
O00909	ADP-ribosylation factor 1	*Symbiodinium*	down
Q23716	Elongation factor 2	*Symbiodinium*	down
O13614	40S ribosomal protein S10-B	*Symbiodinium*	down
O09452	Glyceraldehyde-3-phosphate dehydrogenase, chloroplastic	*Symbiodinium*	down
P53476	Actin	*Symbiodinium*	down
Q01CH5	40S ribosomal protein SA	*Symbiodinium*	down
P93099	60S ribosomal protein L13a	*Symbiodinium*	down
Q6ITB9	Mulgin-3	coral	down
Q9FJX2	60S ribosomal protein L26-2	*Symbiodinium*	down
Q54Z26	Serine hydroxymethyltransferase 1	*Symbiodinium*	down
Q06964	Cysteine proteinase 3	*Symbiodinium*	down
Q7SIB7	Phosphoglycerate kinase 1	*Symbiodinium*	down
Q03604	Ribonucleoside-diphosphate reductase	*Symbiodinium*	down
Q43157	Ribulose-phosphate 3-epimerase, chloroplastic	*Symbiodinium*	down
Q8I4V2	Pre-mRNA-splicing factor PFL2310w	*Symbiodinium*	down

RCP4.5

P41351	Tubulin alpha chain	coral	up
P41352	Tubulin beta chain	coral	up
Q04634	Elongation factor 1-alpha	coral	up
P06147	40S ribosomal protein S12	coral	up
P24119	60S ribosomal protein L11	coral	up
P33190	Ubiquitin-60S ribosomal protein L40	coral	up
Q00454	60S ribosomal protein L27a	coral	up
Q8VHE6	Dynein heavy chain 5, axonemal	coral	up
Q3SEK2	Caltractin ICL1f	coral	up
Q23DE3	40S ribosomal protein S3a	other	up
P24050	40S ribosomal protein S5	coral	up
P24166	Glyceraldehyde-3-phosphate dehydrogenase	other	up
Q91XQ0	Dynein heavy chain 8, axonemal	coral	up
P0CG82	Polyubiquitin	coral	up
Q6XIM8	40S ribosomal protein S15a	other	up
P35685	60S ribosomal protein L7a	other	up
Q9U332	60S ribosomal protein L31	other	up
Q54J69	60S ribosomal protein L10	other	up
Q8VYF1	60S ribosomal protein L15-2	other	up
P62975	Ubiquitin	coral	up
Q98SC7	40S ribosomal protein S29A	other	up
P02598	Calmodulin	coral	up
O16797	60S ribosomal protein L3	coral	up
O15631	40S ribosomal protein S19	other	up
O43992	40S ribosomal protein S2	other	up
P49215	40S ribosomal protein S17	coral	up
Q95V32	40S ribosomal protein S6	coral	up
P25998	60S ribosomal protein L8	coral	up
Q54X53	60S ribosomal protein L21	other	up
P14223	Fructose-bisphosphate aldolase	coral	up

P59696	Transposase for insertion sequence element IS200	other	down
P11941	Lysozyme C II	coral	down
Q92038	Acyl-CoA desaturase	coral	down
P26686	Serine-arginine protein 55	coral	down
P56941	Niemann-Pick C1 protein	coral	down
Q6GL39	WD repeat-containing protein 82	coral	down
Q04671	P protein	coral	down
Q9W6T7	Presenilin-1	coral	down
A8WHP3	Sodium/glucose cotransporter 4	coral	down
Q86SK9	Stearoyl-CoA desaturase 5	coral	down
P47990	Xanthine dehydrogenase/oxidase	coral	down
Q9WVT6	Carbonic anhydrase 14	coral	down
Q28DT7	Polycomb protein eed	coral	down
P35875	Poly [ADP-ribose] polymerase	*Symbiodinium*	down
P25851	Fructose-1,6-bisphosphatase, chloroplastic	coral	down
Q62233	Homeobox protein SIX3	coral	down
P14749	Alpha-galactosidase	coral	down
Q02384	Son of sevenless homolog 2	coral	down
O70196	Prolyl endopeptidase	coral	down
Q61493	DNA polymerase zeta catalytic subunit	coral	down
Q9HCN4	GPN-loop GTPase 1	coral	down
Q8CGC7	Bifunctional aminoacyl-tRNA synthetase	coral	down
P59668	Delta(12) fatty acid desaturase	*Symbiodinium*	down
B8BVB6	Arginine biosynthesis bifunctional protein ArgJ, mitochondrial	*Symbiodinium*	down
Q9UL36	Zinc finger protein 236	coral	down
Q8VE38	Oxidoreductase NAD-binding domain-containing protein 1	coral	down
Q8N4A0	Polypeptide N-acetylgalactosaminyltransferase 4	coral	down
Q6YP21	Kynurenine--oxoglutarate transaminase 3	coral	down
Q5M7N8	Cysteinyl-tRNA synthetase, cytoplasmic	coral	down
Q5H9U9	Probable ATP-dependent RNA helicase DDX60-like	coral	down

RCP8.5

Q9U6Y4	GFP-like fluorescent chromoprotein FP538	coral	up
P17336	Catalase	coral	up
Q9VEG6	Chorion peroxidase	coral	up
Q58A42	Protein DD3-3	coral	up
P38650	Cytoplasmic dynein 1 heavy chain 1	coral	up
Q0E2Y1	(6-4)DNA photolyase	coral	up
A1KZ92	Peroxidasin-like protein	coral	up
P38659	Protein disulfide-isomerase A4	coral	up
Q4UJ69	Cytochrome c oxidase subunit 1	*Symbiodinium*	up
O08721	Netrin receptor UNC5A	coral	up
Q9Z0V5	Peroxiredoxin-4	coral	up
C5NSL2	Bandaporin	coral	up
Q8VHE6	Dynein heavy chain 5, axonemal	coral	up
Q8R4E0	Interferon regulatory factor 2	coral	up
P58912	Toxin PsTX-60B	coral	up
Q923I8	Cryptochrome-2	coral	up
Q63HN8	E3 ubiquitin-protein ligase RNF213	coral	up
A7SK48	Eukaryotic translation initiation factor 3 subunit A	coral	up
Q9BXC9	Bardet-Biedl syndrome 2 protein	coral	up
P48056	Sodium- and chloride-dependent betaine transporter	coral	up
P38657	Protein disulfide-isomerase A3	coral	up
P22079	Lactoperoxidase	coral	up
O88572	Low-density lipoprotein receptor-related protein 6	coral	up
Q9Z0Y8	Voltage-dependent T-type calcium channel subunit alpha-1I	coral	up
Q80U96	Exportin-1	coral	up
P32874	Acetyl-CoA carboxylase, mitochondrial	*Symbiodinium*	up
Q6DDT1	Inositol-3-phosphate synthase 1-B	coral	up
Q7ZY08	Ubiquitin-conjugating enzyme E2 T	coral	up
Q29041	Ficolin-2	coral	up
Q14669	Probable E3 ubiquitin-protein ligase TRIP12	coral	up
P59696	Transposase for insertion sequence element IS200	other	down
Q8UWA5	Carbonic anhydrase 2	coral	down
P11941	Lysozyme C II	coral	down

Q9NB32	T-complex protein 1 subunit delta	coral	down
Q9TT94	Acyl-CoA desaturase	coral	down
Q5RBI5	Splicing factor 3B subunit 3	coral	down
P62498	Eukaryotic peptide chain release factor subunit 1	coral	down
Q86SK9	Stearoyl-CoA desaturase 5	coral	down
Q9EPW2	Krueppel-like factor 15	coral	down
P11960	2-oxoisovalerate dehydrogenase subunit alpha, mitochondrial	coral	down
Q6ITB9	Mulgin-3	coral	down
P10079	Fibropellin-1	coral	down
Q564G3	Sphingolipid delta(4)-desaturase/C4-hydroxylase DES2	*Symbiodinium*	down
Q4V8T0	Inositol oxygenase	coral	down
Q96T37	Putative RNA-binding protein 15	coral	down
Q501J6	Probable ATP-dependent RNA helicase DDX17	coral	down
P11029	Acetyl-CoA carboxylase	coral	down
Q9QW30	Neurogenic locus notch homolog protein 2	coral	down
Q9NR30	Nucleolar RNA helicase 2	coral	down
Q9DE27	RuvB-like 2	coral	down
Q5R5F1	U4/U6 small nuclear ribonucleoprotein Prp3	coral	down
Q9D658	Protein tyrosine phosphatase type IVA 3	coral	down
O70196	Prolyl endopeptidase	coral	down
Q9JJZ4	Ubiquitin-conjugating enzyme E2 J1	coral	down
P54774	Cell division cycle protein 48 homolog	coral	down
Q5FY69	Sodium/glucose cotransporter 5	coral	down
Q54SA7	Probable splicing factor 3B subunit 3	coral	down
Q9Y796	Glyceraldehyde-3-phosphate dehydrogenase	*Symbiodinium*	down
P83606	Kunitz-type serine protease inhibitor 6	coral	down
A8WHP3	Sodium/glucose cotransporter 4	coral	down
