# Supplementary material for: Transcriptomic Changes in Coral Holobionts Provide Insights into Physiological Challenges of Future Climate and Ocean Change
Source: PLoS One. 2015 Oct 28;10(10):e0139223. doi: 10.1371/journal.pone.0139223 (PMC4624983; doi:10.1371/journal.pone.0139223)
Supplement: S2 Table — 13 genes were arbitrarily chosen based on either high up or down regulation in treatment versus present day conditions and a pool of 4 candidate reference genes. (PDF) [file pone.0139223.s007.pdf]

S2 Table. List of candidate genes used in qPCR expression analysis

Name	Product size (bp)	Forward Primer 5'-3'	Reverse Primer 5'-3'
Acyl CoA	100	TGCAAAGCAAATCATGAATGCAT	CAAGCTCCTGACCCTGCTGTT
Carbonic Anhydrase	100	TCTTTTTCAGACGCCGCAGAA	ACAACCCCAACTTGTCTGGAATC
vitellogenin	250	AAGTAAATGGCACATCTGATGACTG	CAGTTGTTGTACATTCTTGGCTCAT
catalase	126	ATTTCATCACACTGCGTCCAGAGACC	TGTCGTTCACCAGTTTGAAGGTGTGGC
E3 ubiquitin-protein ligase	100	ACTTTTGGAGTGTCCGGTTTGTC	GACAACGTCCGTCAAACATCGT
calmodulin	134	GGCGTTGGTCAGACTCTCTC	CAAACGCAAGAGAAGCATCA
cytoskeletal actin	156	TTCATGATGGAGTTGTAGCAGGTTT	AAAGAAAAGCTCTGCTATGTTGCTC
GFP	100	TGTTGACCGCAAACTGGATGTAA	ACGTCAGGCGACCAAAGGTT
Ficolin 2	100	TCGAGTGGTTCTTGTGCAATGA	ATGCGTGTGGTCCACTAAGGTACAG
Kelch like protein 17	115	GCTCATAGGGACAACACAAGTCCAT	AGTCATTGACGGCACTCTTTATGC
Mulgin 3	100	CCAGTATCTGGCTGGGAGGAA	TTCTTTTGGCAGCAGGTGTTGT
HSP 70	100	GTCGCTCTCAATCCATCAAATACT	GTCTCCCACCTTCGCTTACG
**Candidate reference genes**			
Beta-actin	200	CTGATGGACAGGTCATCACCAT	CTCGTGGATACCAGCAGATTCC
Adeno-HomoCyase	66	CCTTGGATGTGCTATGGGTCA	GCCAAGACCTGGTTGGTGAA
Ribosomal protein L7	232	GGAAAGGTCTCCAAACAGCGCACTGCC	CGCGGTTTCCATGATCACCGCCTTCC
Poly(a) binding protein	167	AATGGCGTCTCTATACGTTGG	CGTGAGCTGGTTGTTGGAA
