# Supplementary material for: Transcriptomic Changes in Coral Holobionts Provide Insights into Physiological Challenges of Future Climate and Ocean Change
Source: PLoS One. 2015 Oct 28;10(10):e0139223. doi: 10.1371/journal.pone.0139223 (PMC4624983; doi:10.1371/journal.pone.0139223)
Supplement: S3 Table — The UniProt IDs and descriptions of genes involved in cellular processes for A. millepora holobiont exposed to increased temperature and pCO2 levels predicted by the RPC 8.5 scenario. (PDF) [file pone.0139223.s008.pdf]

S3 Table The UniProt IDs and descriptions of genes involved in cellular processes for *A. millepora* holobiont exposed to increased temperature and pCO_2 levels predicted by the Representative Concentration pathway 8.5 scenario.

UniProt ID	Hit description	Taxonomic origin	Expression direction
Ion transport			
Q9Z0Y8	Voltage-dependent T-type calcium channel subunit alpha-1	coral	up
Q24270	Voltage-dependent calcium channel type D subunit alpha-1	coral	up
P91645	Voltage-dependent calcium channel type A subunit alpha-1	coral	up
P27732	Voltage-dependent L-type calcium channel subunit alpha-1D	coral	up
Q25452	Muscle calcium channel subunit alpha-1	coral	up
O42398	Voltage-dependent L-type calcium channel subunit alpha-1S	coral	up
Q9XES1	Calcium-transporting ATPase 4, endoplasmic reticulum-type	coral	down
P48768	Sodium/calcium exchanger 2	Symbiodinium	down
P70549	Sodium/calcium exchanger 3	Symbiodinium	down
O59768	Vacuolar calcium ion transporter	Symbiodinium	down
Q01728	Sodium/calcium exchanger 1	Symbiodinium	down
O60312	Probable phospholipid-transporting ATPase VA	coral	up
Q26976	V-type proton ATPase subunit B	Symbiodinium	down
O43520	Probable phospholipid-transporting ATPase IC	coral	up
P31400	V-type proton ATPase catalytic subunit A	coral	down
P10719	ATP synthase subunit beta, mitochondrial	Symbiodinium	up
Q4U116	Electrogenic sodium bicarbonate cotransporter 1 (SLC4A4)	coral	down
Q6Q760	Sodium leak channel non-selective protein	coral	up
O17185	Two pore potassium channel protein sup-9	coral	down
Q63472	Potassium voltage-gated channel subfamily H member 1	coral	down
P17659	Potassium voltage-gated channel subfamily A member 6	other	down
Q9Z258	Potassium channel subfamily T member 1	coral	down
P06686	Sodium/potassium-transporting ATPase subunit alpha-2	coral	down
Q5REV9	Sodium-dependent phosphate transport protein 2B (SLC34A2)	coral	down
P48764	Sodium/hydrogen exchanger 3 (SLC9A3)	coral	up
ABC transporters			
Q9FJH6	ABC transporter F family member 1	coral	down
Q84M24	ABC transporter A family member 1	coral	down
P54718	Uncharacterized ABC transporter ATP-binding protein YfiB	other	down
Q8LPJ4	ABC transporter E family member 2	Symbiodinium	down
Q8T6J2	ABC transporter A family member 5	Symbiodinium	up
Q8H0V6	ABC transporter F family member 3	Symbiodinium	up
O53204	Uncharacterized ABC transporter ATP-binding protein Rv2477c/MT2552	Symbiodinium	up
Carbonic Anhydrase			
Q8UWA5	Carbonic anhydrase 2	coral	down
Q27504	Carbonic anhydrase 3	coral	down
Q8VHB5	Carbonic anhydrase 9	coral	down
Q9WVT6	Carbonic anhydrase 14	coral	down
P83299	Carbonic anhydrase 1	coral	down
P48284	Carbonic anhydrase 4	coral	down
Q5BCC5	Carbonic anhydrase	Symbiodinium	up
Cell death/immunity			
Q8WXG1	Radical S-adenosyl methionine domain-containing protein 2	coral	down
Q6NZ06	Interleukin enhancer-binding factor 2 homolog	coral	down
Q10471	Polypeptide N-acetylgalactosaminyltransferase 2	coral	down
P11941	Lysozyme C II	coral	down
O15033	Protein KIAA0317	coral	down
Q29041	Ficolin-2	coral	up
Q9VQQ9	Exocyst complex component 2	coral	up
Q8R5F7	Interferon-induced helicase C domain-containing protein 1	coral	up
Q5ZJL9	SAM domain and HD domain-containing protein 1	coral	up
P04839	Cytochrome b-245 heavy chain	coral	up
Q3UG20	Histone-lysine N-methyltransferase MLL5	coral	up
Q86FQ0	Cytolysin Src-1	coral	up
P58912	Toxin PsTX-60B	coral	up
Q02817	Mucin-2	coral	up
Q60803	TNF receptor-associated factor 3	coral	up
Q29042	Ficolin-1	coral	up
Q63772	Growth arrest-specific protein 6	coral	up
P25092	Heat-stable enterotoxin receptor	coral	up
P19109	ATP-dependent RNA helicase p62	other	up
Q93109	Equinatoxin-5	other	up
Oxidative stress			
P09933	Thyroid peroxidase	coral	down
Q23490	Peroxidase mlt-7	coral	down
P17336	Catalase	coral	up
Q9Z0V5	Peroxiredoxin-4	coral	up
Q9VEG6	Chorion peroxidase	coral	up
O61235	Catalase-2	coral	up

Q27487	Peroxisomal catalase 1	coral	up
P11247	Myeloperoxidase	coral	up
O16025	Allene oxide synthase-lipoxygenase protein	coral	up
P22079	Lactoperoxidase	coral	up
O48646	Probable phospholipid hydroperoxide glutathione peroxidase 6, mitochondrial	coral	up
Q4PD66	Putative heme-binding peroxidase	Symbiodinium	up
Q42592	L-ascorbate peroxidase S, chloroplastic/mitochondrial	Symbiodinium	up
Molecular chaperones			
Q498R3	DnaJ homolog subfamily C member 10	coral	up
Q8NFJ9	Bardet-Biedl syndrome 1 protein	coral	up
Q99MH9	Bardet-Biedl syndrome 2 protein homolog	coral	up
Q8K0U4	Heat shock 70 kDa protein 12A	coral	up
Q9JLC8	Sacsin	coral	up
Q5RGU1	Chaperone activity of bc1 complex-like, mitochondrial	coral	up
Q54HY8	Probable mitochondrial chaperone BCS1-A	other	down
Q6F2Y7	Heat shock protein 101	Symbiodinium	down
P19208	Heat shock 70 kDa protein C	Symbiodinium	down
Q9USI5	Heat shock protein sti1 homolog	Symbiodinium	down
Cell homeostasis			
Q63120	Canalicular multispecific organic anion transporter 1	coral	down
P55011	Solute carrier family 12 member 2	coral	down
Q04447	Creatine kinase B-type	coral	down
Q920H8	Hephaestin	coral	down
Q5ZIH2	Vacuolar fusion protein MON1 homolog A	coral	down
Q8JZQ2	AFG3-like protein 2	coral	down
Q5NVA2	Thioredoxin reductase 1, cytoplasmic	coral	down
Q9FNE2	Glutaredoxin-C2	coral	down
A6H784	Protein SCO2 homolog, mitochondrial	coral	down
A7RQM5	ATPase ASNA1 homolog	coral	down
P56941	Niemann-Pick C1 protein	coral	down
Q6PBM1	Glutaredoxin-related protein 5, mitochondrial	coral	down
Q86WG5	Myotubularin-related protein 13	coral	up
Q9ESR9	ATP-binding cassette sub-family A member 2	coral	up
Q5E9M9	Mitochondrial Rho GTPase 2	coral	up
Q9D8X1	Copper homeostasis protein cutC homolog	coral	up
O95881	Thioredoxin domain-containing protein 12	coral	up
Q8NBS9	Thioredoxin domain-containing protein 5	Symbiodinium	down
P10719	ATP synthase subunit beta, mitochondrial	Symbiodinium	up
O95477	ATP-binding cassette sub-family A member 1	Symbiodinium	up
P83877	Thioredoxin-like protein 4A	Symbiodinium	up
Response to ER stress			
Q32LH7	Cytochrome b5 reductase 4	coral	down
P54399	Protein disulfide-isomerase	coral	up
O22263	Protein disulfide-isomerase like 2-1	coral	up
Q67IX6	Protein disulfide isomerase-like 1-4	coral	up
Q17967	Protein disulfide-isomerase 1	coral	up
P38657	Protein disulfide-isomerase A3	coral	up
Q29RV1	Protein disulfide-isomerase A4	coral	up
O94726	ER degradation-enhancing alpha-mannosidase-like protein 1	coral	up
P09103	Protein disulfide-isomerase	Symbiodinium	up
P34329	Probable protein disulfide-isomerase A4	Symbiodinium	up
Calcium signaling/homeostasis			
Q64143	Phosphatidylinositol 3-kinase regulatory subunit gamma	coral	down
Q8CHJ1	Phosphatidylinositol glycan anchor biosynthesis class U protein	coral	down
P29994	Inositol 1,4,5-trisphosphate receptor type 1	coral	up
Q63269	Inositol 1,4,5-trisphosphate receptor type 3	coral	up
Q14571	Inositol 1,4,5-trisphosphate receptor type 2	coral	up
P29993	Inositol 1,4,5-trisphosphate receptor	coral	up
A2A891	Calmodulin-binding transcription activator 1	coral	up
Q6DEH3	Calcium/calmodulin-dependent protein kinase type II delta 1 chain	coral	up
Q9Y2I7	1-phosphatidylinositol-3-phosphate 5-kinase	coral	up
P51432	1-phosphatidylinositol-4,5-bisphosphate phosphodiesterase beta-3	coral	up
A4IID4	Phosphatidylinositol 4-kinase beta	coral	up
Q22908	Ras and EF-hand domain-containing protein homolog	coral	up
A0JP43	EF-hand calcium-binding domain-containing protein 5	coral	up
A2ARV4	Low-density lipoprotein receptor-related protein 2	coral	up
Q32TF8	EF-hand domain-containing family member C2	other	down
O96102	Calmodulin	other	up
Wnt Signalling			
P33945	Protein Wnt-5b	coral	down
O42280	Protein Wnt-9a	coral	up
P51028	Protein Wnt-8a	coral	up

P56706	Protein Wnt-7b	coral	up
Q8R2H4	Kelch-like protein 12	coral	up
Q91VN0	Low-density lipoprotein receptor-related protein 5	coral	up
O88572	Low-density lipoprotein receptor-related protein 6	coral	up
P67963	Casein kinase I isoform alpha	coral	up
Q9Y283	Inversin	coral	up
Q9U2Q9	Glycogen synthase kinase-3	Symbiodinium	down
Notch signaling			
Q07008	Neurogenic locus notch homolog protein 1	coral	down
Q9QW30	Neurogenic locus notch homolog protein 2	coral	down
Q61982	Neurogenic locus notch homolog protein 3	coral	down
P31695	Neurogenic locus notch homolog protein 4	coral	down
P07207	Neurogenic locus Notch protein	coral	down
Circadian clock			
Q923I8	Cryptochrome-2	coral	up
Q6ZZY0	Cryptochrome-1	coral	up
Q9QZQ0	Neuronal PAS domain-containing protein 3	coral	up
Q60520	Paired amphipathic helix protein Sin3a	coral	up
Q8R1B8	Nuclear receptor ROR-beta	coral	up
Q9QXZ7	Photoreceptor-specific nuclear receptor	coral	up
Extra cellular matrix			
Q04906	Bone morphogenetic protein 6	coral	down
P08122	Collagen alpha-2(IV) chain	coral	down
Q4LDE5	Sushi, von Willebrand factor type A, EGF and pentraxin domain-containing protein 1	coral	down
P10079	Fibropellin-1	coral	down
P00747	Plasminogen	coral	down
A1KZ92	Peroxidasin-like protein	coral	up
Q5UQ50	Collagen-like protein 6	coral	up
P23206	Collagen alpha-1(X) chain	coral	up
P53420	Collagen alpha-4(IV) chain	coral	up
Q5UQ13	Collagen-like protein 2	coral	up
P29400	Collagen alpha-5(IV) chain	coral	up
Q5UQ50	Collagen-like protein 6	coral	up
P29119	Furin-1	coral	up
Q06441	Thrombospondin-4	coral	up
P07996	Thrombospondin-1	coral	up
O57382	Tolloid-like protein 2	coral	up
DNA damage/repair			
P54275	DNA mismatch repair protein Msh2	coral	down
O15164	Transcription intermediary factor 1-alpha	coral	down
P23571	DNA-3-methyladenine glycosylase (Fragment)	coral	down
Q6C3X6	RuvB-like helicase 2	coral	down
Q9DEI1	DNA-dependent protein kinase catalytic subunit	coral	down
Q8IRG6	FACT complex subunit spt16	coral	down
Q5KND8	RuvB-like helicase 2	coral	down
Q9DBR1	5'-3' exoribonuclease 2	coral	down
P54276	DNA mismatch repair protein Msh6	coral	up
O14802	DNA-directed RNA polymerase III subunit RPC1	coral	up
P97679	DNA mismatch repair protein Mlh1	coral	up
P43247	DNA mismatch repair protein Msh2	coral	up
P36601	DNA repair protein rhp51	coral	up
Q92889	DNA repair endonuclease XPF	coral	up
O35923	Breast cancer type 2 susceptibility protein homolog	coral	up
Q61493	DNA polymerase zeta catalytic subunit	coral	up
Q02870	DNA excision repair protein haywire	coral	up
P78549	Endonuclease III-like protein 1	coral	up
Q9DE46	DNA polymerase alpha catalytic subunit	coral	up
Q80TP3	E3 ubiquitin-protein ligase UBR5	coral	up
Q61880	Meiotic recombination protein DMC1/LIM15 homolog	coral	up
Q4G056	Tyrosyl-DNA phosphodiesterase 1	coral	up
Q8BKX6	Serine/threonine-protein kinase SMG1	coral	up
Q5FVP2	SOSS complex subunit B2	coral	up
Q5U4C9	Dual specificity tyrosine-phosphorylation-regulated kinase 2	coral	up
Q96T88	E3 ubiquitin-protein ligase UHRF1	coral	up
Q9NVI1	Fanconi anemia group I protein	coral	up
P43352	DNA repair protein RAD52 homolog	coral	up
Q5XHZ9	Thyroid receptor-interacting protein 13	coral	up
Q8K368	Fanconi anemia group I protein homolog	coral	up
Q0E2Y1	(6-4)DNA photolyase	coral	up
P51954	Serine/threonine-protein kinase Nek1	coral	up
O54747	DNA polymerase delta catalytic subunit	coral	up
Q9P2Y5	UV radiation resistance-associated gene protein	coral	up
Q99MT2	MutS protein homolog 4	coral	up

Q9LEF5	FACT complex subunit SSRP1	coral	up
Q6ZQF0	DNA topoisomerase 2-binding protein 1	coral	up
O15457	MutS protein homolog 4	other	down
Q5DU05	Centrosomal protein of 164 kDa	Symbiodinium	down
Q1XDF4	ATP-dependent Clp protease ATP-binding subunit clpA homolog	Symbiodinium	down
P29295	Casein kinase I homolog HRR25	Symbiodinium	up
Q5BBV9	RuvB-like helicase 1	Symbiodinium	up
Q99PK0	Pre-mRNA-splicing factor SYF1	Symbiodinium	up
Q59LR2	Serine/threonine-protein kinase MEC1	Symbiodinium	up
Q9SL02	DNA repair protein RAD50	Symbiodinium	up
Proteolysis			
Q63570	26S protease regulatory subunit 6B	coral	down
P19205	Acylamino-acid-releasing enzyme	coral	down
Q8JZQ2	AFG3-like protein 2	coral	down
Q9NR09	Baculoviral IAP repeat-containing protein 6	coral	down
Q9JLG8	Calpain-15	coral	down
Q9JJ31	Cullin-5	coral	down
Q9D1A2	Cytosolic non-specific dipeptidase	coral	down
Q6I6G8	E3 ubiquitin-protein ligase HECW2	coral	down
Q6GNY1	E3 ubiquitin-protein ligase mib1	coral	down
Q6E2N3	E3 ubiquitin-protein ligase TRIM33	coral	down
P42785	Lysosomal Pro-X carboxypeptidase	coral	down
Q7ZVX6	NEDD8-activating enzyme E1 catalytic subunit	coral	down
P79953	Ovochymase-2	coral	down
P26262	Plasma kallikrein	coral	down
A7SXZ6	Probable tRNA threonylcarbamoyladenosine biosynthesis protein osgep	coral	down
P34676	Prolyl carboxy peptidase like protein 5	coral	down
O70196	Prolyl endopeptidase	coral	down
Q9Z1K5	Protein ariadne-1 homolog	coral	down
O76924	Protein ariadne-2	coral	down
O15033	Protein KIAA0317	coral	down
Q9HB40	Retinoid-inducible serine carboxypeptidase	coral	down
Q5U405	Transmembrane protease serine 13	coral	down
Q7Z410	Transmembrane protease serine 9	coral	down
Q5R761	U4/U6.U5 tri-snRNP-associated protein 2	coral	down
Q5RCD3	Ubiquitin carboxyl-terminal hydrolase 4	coral	down
Q5R407	Ubiquitin carboxyl-terminal hydrolase 5	coral	down
Q70EK9	Ubiquitin carboxyl-terminal hydrolase 51	coral	down
Q9JJZ4	Ubiquitin-conjugating enzyme E2 J1	coral	down
A0PJN4	Ubiquitin-conjugating enzyme E2Q-like protein 1	coral	down
Q9D906	Ubiquitin-like modifier-activating enzyme ATG7	coral	down
Q9YMP9	Viral cathepsin	coral	down
Q8CEG8	Ubiquitin carboxyl-terminal hydrolase 27	coral	down
Q7KN62	Transitional endoplasmic reticulum ATPase TER94	coral	down
P28840	Neuroendocrine convertase 1	coral	down
P97435	Enteropeptidase	coral	down
Q8BU03	Periodic tryptophan protein 2 homolog	coral	down
O15072	A disintegrin and metalloproteinase with thrombospondin motifs 3	coral	up
Q9UKP4	A disintegrin and metalloproteinase with thrombospondin motifs 7	coral	up
A4IHP4	Cullin-3	coral	up
Q0P4M4	Cytosolic carboxypeptidase 2	coral	up
Q96MI9	Cytosolic carboxypeptidase 4	coral	up
Q8CF97	Deubiquitinating protein VCIP135	coral	up
O70260	E3 SUMO protein ligase PIAS3	coral	up
Q69ZR2	E3 ubiquitin-protein ligase HECTD1	coral	up
Q4U2R1	E3 ubiquitin-protein ligase HERC2	coral	up
Q7TMY8	E3 ubiquitin-protein ligase HUWE1	coral	up
Q6ZQ89	E3 ubiquitin-protein ligase MARCH6	coral	up
Q5ZIJ9	E3 ubiquitin-protein ligase MIB2	coral	up
Q5R7T5	E3 ubiquitin-protein ligase NRDP1	coral	up
Q9R1A8	E3 ubiquitin-protein ligase RFWD2	coral	up
Q2TL32	E3 ubiquitin-protein ligase UBR4	coral	up
Q80TP3	E3 ubiquitin-protein ligase UBR5	coral	up
Q7TPK1	E3 ubiquitin-protein ligase UHRF1	coral	up
P42893	Endothelin-converting enzyme 1	coral	up
Q8VBV4	F-box/WD repeat-containing protein 7	coral	up
Q9R1K5	Fizzy-related protein homolog	coral	up
Q9CXY9	GPI-anchor transamidase	coral	up
P35559	Insulin-degrading enzyme	coral	up
Q1L5Z9	LON peptidase N-terminal domain and RING finger protein 2	coral	up
Q9D4H7	LON peptidase N-terminal domain and RING finger protein 3	coral	up
Q9Y3Q0	N-acetylated-alpha-linked acidic dipeptidase 2	coral	up
Q9UQQ1	N-acetylated-alpha-linked acidic dipeptidase-like protein	coral	up
Q8R554	OTU domain-containing protein 7A	coral	up
Q5K2P9	Polyserase-2	coral	up
Q14669	Probable E3 ubiquitin-protein ligase TRIP12	coral	up

P70398	Probable ubiquitin carboxyl-terminal hydrolase FAF-X	coral	up
Q8VDW4	Probable ubiquitin-conjugating enzyme E2 W	coral	up
Q61139	Proprotein convertase subtilisin/kexin type 7	coral	up
Q16651	Prostasin	coral	up
Q8T4E1	Putative GPI-anchor transamidase	coral	up
Q9HBA9	Putative N-acetylated-alpha-linked acidic dipeptidase	coral	up
Q9FHW7	SKP1-like protein 1B	coral	up
A0JMG1	Speckle-type POZ protein-like B	coral	up
Q9Z1F9	SUMO-activating enzyme subunit 2	coral	up
O54928	Suppressor of cytokine signaling 5	coral	up
Q9UPU5	Ubiquitin carboxyl-terminal hydrolase 24	coral	up
Q70CQ4	Ubiquitin carboxyl-terminal hydrolase 31	coral	up
Q8NFA0	Ubiquitin carboxyl-terminal hydrolase 32	coral	up
Q6ZQ93	Ubiquitin carboxyl-terminal hydrolase 34	coral	up
Q8BUM9	Ubiquitin carboxyl-terminal hydrolase 43	coral	up
P52483	Ubiquitin-conjugating enzyme E2 E3	coral	up
Q9NPD8	Ubiquitin-conjugating enzyme E2 T	coral	up
P55115	Zinc metalloproteinase nas-15	coral	up
Q8VZS9	Protein FIZZY-RELATED 1	other	down
Q9DBI0	Transmembrane protease serine 6	other	up
P46472	26S protease regulatory subunit 7	Symbiodinium	down
Q1XDF9	ATP-dependent zinc metalloprotease FtsH	Symbiodinium	down
Q8H0W1	Chloroplast processing peptidase	Symbiodinium	down
P22412	Dipeptidase 1	Symbiodinium	down
Q7KUT2	Lon protease homolog, mitochondrial	Symbiodinium	down
B8J198	Lon protease	Symbiodinium	down
Q8L3Z8	Protein FIZZY-RELATED 2	Symbiodinium	down
Q8LPL5	Protein FIZZY-RELATED 3	Symbiodinium	down
Q42384	Protein pleiotropic regulatory locus 1	Symbiodinium	down
Q9FF66	Ubiquitin-conjugating enzyme E2 22	Symbiodinium	down
Q9LJL3	Presequence protease 1, chloroplastic/mitochondrial	Symbiodinium	down
Q5PQY6	Lon protease homolog 2, peroxisomal	Symbiodinium	down
Q8VY06	Presequence protease 2, chloroplastic/mitochondrial	Symbiodinium	down
Q4FZT9	26S proteasome non-ATPase regulatory subunit 2	Symbiodinium	up
P72991	ATP-dependent zinc metalloprotease FtsH 3	Symbiodinium	up
A6QBN8	ATP-dependent zinc metalloprotease FtsH	Symbiodinium	up
Q6GPU1	Cysteine protease ATG4A	Symbiodinium	up
O74445	Probable 26S protease subunit rpt4	Symbiodinium	up
Q6J1Y9	Ubiquitin carboxyl-terminal hydrolase 19	Symbiodinium	up
Cytoskeletal interactions			
P27619	Dynamin	coral	down
P46824	Kinesin light chain	coral	down
Q7KN62	Transitional endoplasmic reticulum ATPase TER94	coral	down
P52275	Tubulin beta-2 chain	coral	down
O73787	Gamma-tubulin complex component 3 homolog	coral	down
Q7TSG1	Centrosomal protein of 120 kDa	coral	up
P38650	Cytoplasmic dynein 1 heavy chain 1	coral	up
Q27802	Cytoplasmic dynein 2 heavy chain 1	coral	up
Q14008	Cytoskeleton-associated protein 5	coral	up
Q6PCJ1	Dynactin subunit 1	coral	up
P23098	Dynein beta chain, ciliary	coral	up
Q9P2D7	Dynein heavy chain 1, axonemal	coral	up
Q8IVF4	Dynein heavy chain 10, axonemal	coral	up
Q96DT5	Dynein heavy chain 11, axonemal	coral	up
Q923J6	Dynein heavy chain 12, axonemal	coral	up
Q9UFH2	Dynein heavy chain 17, axonemal	coral	up
Q9P225	Dynein heavy chain 2, axonemal	coral	up
Q8TD57	Dynein heavy chain 3, axonemal	coral	up
Q8TE73	Dynein heavy chain 5, axonemal	coral	up
Q9C0G6	Dynein heavy chain 6, axonemal	coral	up
Q8WXX0	Dynein heavy chain 7, axonemal	coral	up
Q91XQ0	Dynein heavy chain 8, axonemal	coral	up
Q9NYC9	Dynein heavy chain 9, axonemal	coral	up
P37276	Dynein heavy chain, cytoplasmic	coral	up
Q9TVM2	Exportin-1	coral	up
Q92845	Kinesin-associated protein 3	coral	up
P46871	Kinesin-II 95 kDa subunit	coral	up
Q9GYZ0	Kinesin-like protein KIF15	coral	up
O35787	Kinesin-like protein KIF1C	coral	up
Q9QXL1	Kinesin-like protein KIF21B	coral	up
Q52KG5	Kinesin-like protein KIF26A	coral	up
Q7TNC6	Kinesin-like protein KIF26B	coral	up
P28739	Kinesin-like protein klpA	coral	up
Q63356	Myosin-Ie	coral	up
Q29P71	Myosin-VIIa	coral	up
Q6PIF6	Myosin-VIIb	coral	up

Q5ZMV2	Spindle assembly abnormal protein 6 homolog	coral	up
Q62868	Rho-associated protein kinase 2	coral	up
Q8WQ47	Tubulin alpha chain	coral	up
O44388	Tubulin beta chain	coral	up
Q9D6F9	Tubulin beta-4 chain	coral	up
Q9UJT0	Tubulin epsilon chain	coral	up
Q14008	Cytoskeleton-associated protein 5	coral	up
Q9P2E2	Kinesin-like protein KIF17	other	up
Q9U2Q9	Glycogen synthase kinase-3	Symbiodinium	down
P29295	Casein kinase I homolog HRR25	Symbiodinium	up
Q9QXL2	Kinesin-like protein KIF21A	Symbiodinium	up
P23257	Tubulin gamma-1 chain	Symbiodinium	up
Cell cycle/cell proliferation regulation			
Q8CG48	Structural maintenance of chromosomes protein 2	coral	down
Q6AYI2	Kelch domain-containing protein 3	coral	down
Q9Y236	Oxidative stress-induced growth inhibitor 2	coral	down
Q61127	NGFI-A-binding protein 2	coral	down
Q4V8A2	Cell division cycle protein 27 homolog	coral	down
Q28D84	Histone-lysine N-methyltransferase EZH2	coral	down
Q28DT7	Polycomb protein eed	coral	down
Q641W4	Replication factor C subunit 2	coral	down
Q9DEI1	DNA-dependent protein kinase catalytic subunit	coral	down
P97386	DNA ligase 3	coral	down
Q64213	Splicing factor 1	coral	up
P0C279	Cytoplasmic polyadenylation element-binding protein 1	coral	up
Q61880	Meiotic recombination protein DMC1/LIM15 homolog	coral	up
P38129	Transcription initiation factor TFIID subunit 5	coral	up
Q7TSG1	Centrosomal protein of 120 kDa	coral	up
Q66II3	Growth factor receptor-bound protein 2	coral	up
Q6KCD5	Nipped-B-like protein	coral	up
Q00526	Cyclin-dependent kinase 3	coral	up
P50532	Structural maintenance of chromosomes protein 4	coral	up
P50533	Structural maintenance of chromosomes protein 2	coral	up
Q5ZMV2	Spindle assembly abnormal protein 6 homolog	coral	up
P62296	Abnormal spindle-like microcephaly-associated protein homolog	coral	up
Q8JZP9	GAS2-like protein 1	coral	up
Q8VIG3	Radial spoke head 1 homolog	coral	up
P42128	Forkhead box protein K1	coral	up
Q9U2Q9	Glycogen synthase kinase-3	Symbiodinium	down
P97310	DNA replication licensing factor MCM2	symbiodinium	down
Q80YP0	Cyclin-dependent kinase 3	Symbiodinium	up
P23573	Cell division control protein 2 cognate	Symbiodinium	up
P30665	DNA replication licensing factor MCM4	Symbiodinium	up
Q5DU05	Centrosomal protein of 164 kDa	symbiodinium	up
Q9UW86	Serine/threonine-protein phosphatase PP1	Symbiodinium	up
Metabolism			
P11960	2-oxoisovalerate dehydrogenase subunit alpha, mitochondrial (Fragment)	coral	down
Q8BMF3	NADP-dependent malic enzyme, mitochondrial	coral	down
Q6PEI7	CTP synthase 1	coral	down
Q6P963	Hydroxyacylglutathione hydrolase, mitochondrial	coral	down
Q28FR6	3-hydroxyisobutyryl-CoA hydrolase, mitochondrial	coral	down
P35738	2-oxoisovalerate dehydrogenase subunit beta, mitochondrial	coral	down
Q99KE1	NAD-dependent malic enzyme, mitochondrial	coral	down
Q2KIZ3	Methylmalonyl-CoA epimerase, mitochondrial	coral	down
Q8BHF7	CDP-diacylglycerol--glycerol-3-phosphate 3-phosphatidyltransferase, mitochondrial	coral	down
Q148G5	Proline dehydrogenase 1, mitochondrial	coral	down
Q08D64	ATP-binding cassette sub-family B member 6, mitochondrial	coral	down
Q5ZJ60	3-hydroxyisobutyryl-CoA hydrolase, mitochondrial	coral	down
Q5RFN2	Methylmalonyl-CoA mutase, mitochondrial	coral	down
B1P1W2	Succinate dehydrogenase assembly factor 2, mitochondrial	coral	down
Q3SZI8	Isovaleryl-CoA dehydrogenase, mitochondrial	coral	down
P70079	Creatine kinase U-type, mitochondrial	coral	down
Q8QZS1	3-hydroxyisobutyryl-CoA hydrolase, mitochondrial	coral	down
Q1JPD3	D-2-hydroxyglutarate dehydrogenase, mitochondrial	coral	down
P50671	Cytochrome c oxidase subunit 1 (Fragment)	coral	down
Q96190	Cytochrome c oxidase subunit 2	coral	down
B0U3F2	Glycerol kinase	coral	down
Q9TTS3	Acetyl-CoA carboxylase 1	coral	down
P12785	Fatty acid synthase	coral	down
Q68J42	Hormone-sensitive lipase	coral	down
Q9UH92	Max-like protein X	coral	down
P38652	Phosphoglucomutase-1	coral	down
P79303	UTP--glucose-1-phosphate uridylyltransferase	coral	down
P17625	Glycogen [starch] synthase, liver	coral	down
A6QLU1	Glycerol-3-phosphate dehydrogenase, mitochondrial	coral	up

Q3SY69	Aldehyde dehydrogenase family 1 member L2, mitochondrial	coral	up
Q9C8L4	Hydroxyacylglutathione hydrolase 3, mitochondrial	coral	up
Q8CFA2	Aminomethyltransferase, mitochondrial	coral	up
P11498	Pyruvate carboxylase, mitochondrial	coral	up
P50668	Cytochrome c oxidase subunit 1	other	down
Q9LJL3	Presequence protease 1, chloroplastic/mitochondrial	Symbiodinium	down
Q28DK1	Citrate synthase, mitochondrial	Symbiodinium	down
Q29551	Succinyl-CoA:3-ketoacid-coenzyme A transferase 1, mitochondrial	Symbiodinium	down
Q2W3W0	Fructose-1,6-bisphosphatase class 1	Symbiodinium	down
Q0WM29	Methylmalonate-semialdehyde dehydrogenase [acylating], mitochondrial	Symbiodinium	down
Q55FN7	2-oxoisovalerate dehydrogenase subunit beta, mitochondrial	Symbiodinium	down
P39522	Dihydroxy-acid dehydratase, mitochondrial	Symbiodinium	down
Q27877	Enolase	Symbiodinium	down
Q54RR5	Probable short/branched chain specific acyl-CoA dehydrogenase	Symbiodinium	down
Q6FRR0	Phosphoenolpyruvate carboxykinase [ATP]	Symbiodinium	down
Q8YRU9	Transketolase	Symbiodinium	down
Q94IN5	Pyruvate dehydrogenase [NADP+], mitochondrial	Symbiodinium	up
Q02226	Cytochrome c oxidase subunit 2, mitochondrial (Fragment)	Symbiodinium	up
Q9ZPX5	Succinate dehydrogenase [ubiquinone] flavoprotein subunit 2, mitochondrial	Symbiodinium	up
Q623T0	2-oxoglutarate dehydrogenase, mitochondrial	Symbiodinium	up
